# Phylogenetic and Metabolic Tracking of Gut Microbiota during Perinatal Development

**DOI:** 10.1371/journal.pone.0137347

**Published:** 2015-09-02

**Authors:** Federica Del Chierico, Pamela Vernocchi, Andrea Petrucca, Paola Paci, Susana Fuentes, Giulia Praticò, Giorgio Capuani, Andrea Masotti, Sofia Reddel, Alessandra Russo, Cristina Vallone, Guglielmo Salvatori, Elsa Buffone, Fabrizio Signore, Giuliano Rigon, Andrea Dotta, Alfredo Miccheli, Willem M. de Vos, Bruno Dallapiccola, Lorenza Putignani

**Affiliations:** 1 Unit of Metagenomics, Bambino Gesù Children’s Hospital, IRCCS, Rome, Italy; 2 Interdipartimental Centre for Industrial Research-CIRI-AGRIFOOD, Alma Mater Studiorum, University of Bologna, Bologna, Italy; 3 Department of Diagnostic Science, Sant’Andrea Hospital, Rome, Italy; 4 CNR, Institute of Systems Analysis and Informatics Antonio Ruberti, Rome, Italy; 5 Laboratory of Microbiology, Wageningen University, Wageningen, The Netherlands; 6 Department of Chemistry, Sapienza University, Rome, Italy; 7 Bambino Gesù Children Hospital, IRCCS, Rome, Italy; 8 Department of Obstetrics and Gyneacology, San Camillo Hospital, Rome, Italy; 9 Department of Neonatology, Bambino Gesù Children’s Hospital, IRCCS, Rome, Italy; 10 Department of Neonatology, San Camillo Hospital, Rome, Italy; 11 Departments of Veterinary Biosciences and Bacteriology & Immunology, Helsinki University, Helsinki, Finland; 12 Scientific Directorate, Bambino Gesù Children’s Hospital, IRCCS, Rome, Italy; 13 Unit of Parasitology, Bambino Gesù Children’s Hospital, IRCCS, Rome, Italy; GI Lab, UNITED STATES

## Abstract

The colonization and development of gut microbiota immediately after birth is highly variable and depends on several factors, such as delivery mode and modality of feeding during the first months of life. A cohort of 31 mother and neonate pairs, including 25 at-term caesarean (CS) and 6 vaginally (V) delivered neonates (DNs), were included in this study and 121 meconium/faecal samples were collected at days 1 through 30 following birth. Operational taxonomic units (OTUs) were assessed in 69 stool samples by phylogenetic microarray HITChip and inter- and intra-individual distributions were established by inter-OTUs correlation matrices and OTUs co-occurrence or co-exclusion networks. ^1^H-NMR metabolites were determined in 70 stool samples, PCA analysis was performed on 55 CS DNs samples, and metabolome/OTUs co-correlations were assessed in 45 CS samples, providing an integrated map of the early microbiota OTUs-metabolome. A microbiota “core” of OTUs was identified that was independent of delivery mode and lactation stage, suggesting highly specialized communities that act as seminal colonizers of microbial networks. Correlations among OTUs, metabolites, and OTUs-metabolites revealed metabolic profiles associated with early microbial ecological dynamics, maturation of milk components, and host physiology.

## Introduction

The microbial colonization from birth to adulthood of a healthy human gut is characterized by a dynamic sequence of events that plays a critical role in promoting intestinal homeostasis and stimulating normal immune system (IS) development and response. These functions provide an array of beneficial advantages to the human host, including nutrient processing and metabolism, energy storage, and protection against pathogen colonization [1–9]. The gastrointestinal (GI) microbiota is composed by autochthonous (*i*.*e*., indigenous) and allochthonous (*i*.*e*., transient) microbes [2]. Most pathogens are allochthonous microorganisms. However, some potential pathogens can be considered autochthonous and normally reside unperturbed within the colonic host microbiota until the ecosystem is perturbed and homeostasis is disturbed. Abnormalities in gut microbiome composition and associated changes in IS tolerance have been increasingly linked with early-onset childhood diseases [10]. Pioneering gut constituents and their impact on metabolic pathways in early infancy may have both short- and long-term health-related implications in humans [4,11–14]. However, while gut microbiota in neonates is characterized by high inter-individual variability and uneven distribution of taxa throughout this developmental stage [15–18], it remains unclear what role this variation has in the function and the progression of the microbial community in the very early stages of life [18].

Several studies have shown that both differential exposure of neonates to maternal vaginal, faecal, and skin microbiota due to delivery mode (*i*.*e*., vaginal or C-section) and modality of feeding during the first months of life (*i*.*e*., breastfeeding and/or formula-feeding) accounts for the richness, diversity, and composition of the gut microbial community [1,18–25]. However, little is known regarding the bacterial composition of meconium through to faeces during the first weeks of life [16,26,27]. Studies on meconium bacterial diversity, highlighting the succession of gut microbiota communities, could assist in developing strategies for selecting health-promoting microbiota for preservation throughout the lifespan of the host [28,29]. These microbial communities could be considered targets for therapeutic intervention in several chronic childhood diseases [30–33].

Gut microbiota contributes to the host metabolism through numerous mechanisms, including increased energy harvested from the diet, modulation of lipid metabolism, altered endocrine function, and increased inflammatory response [34,35]. Microbiota metabolic functions are mainly based on the fermentation of available substrates that have escaped digestion in the upper GI tract and flaking enterocytes [36]. In the early days of life, the major sources of carbohydrates for infant gut microbiota are human milk oligosaccharides (HMOSs), endogenous glycans (mucines), undigested monosaccharides and lactose, and, in the case of formula feeding, prebiotics [37]. Complex carbohydrates are principally degraded by *Bifidobacterium*, *Lactobacillus*, and *Bacteroides* (HMOSs and mucin degraders) into small sugars (*e*.*g*., lactose), which are further metabolized by glycolytic microbes, including lactate producing bacteria such as *Streptococcus*, *Staphylococcus*, *Enterococcus*, and Enterobacteria [38].

Thus, a major challenge in the understanding of host–microbiome metabolic interactions, notably the role of gut microbiota in early life, is how it affects an individual’s health programming. Metabolomics may play a pivotal role in understanding the mechanism of infant intestinal development. Such approaches involve the identification and quantification of hundreds of small metabolites from bodily fluids and tissues, providing functional insights into the complex interactions between gut microbes and the host [39]. While several NMR-based metabolomic studies on human adult stool have been performed to evaluate the influence of diet on gut microbiota or the association of dysbiosis with intestinal inflammatory diseases [40–42], metabolomic investigations into the meconium or stool from infants are quite limited [43,44].

The goal of this pilot study was to investigate the chronological succession and network of interactions of gut microbiota operational taxonomic units (OTUs). Correlation maps among OTUs and between OTUs and related metabolites were also determined by identifying NMR-based faecal metabolome profiles during gut microbiota evolution throughout the first thirty days of life. Correlation maps between OTUs and metabolome components allowed us to investigate early phyla-associated metabolic activities, shedding light onto the metabolome framework during early intestinal microbial development.

## Results and Discussion

### Early gut microbiota profiles: punctiform and longitudinal phyla distribution

**Fig 1:**
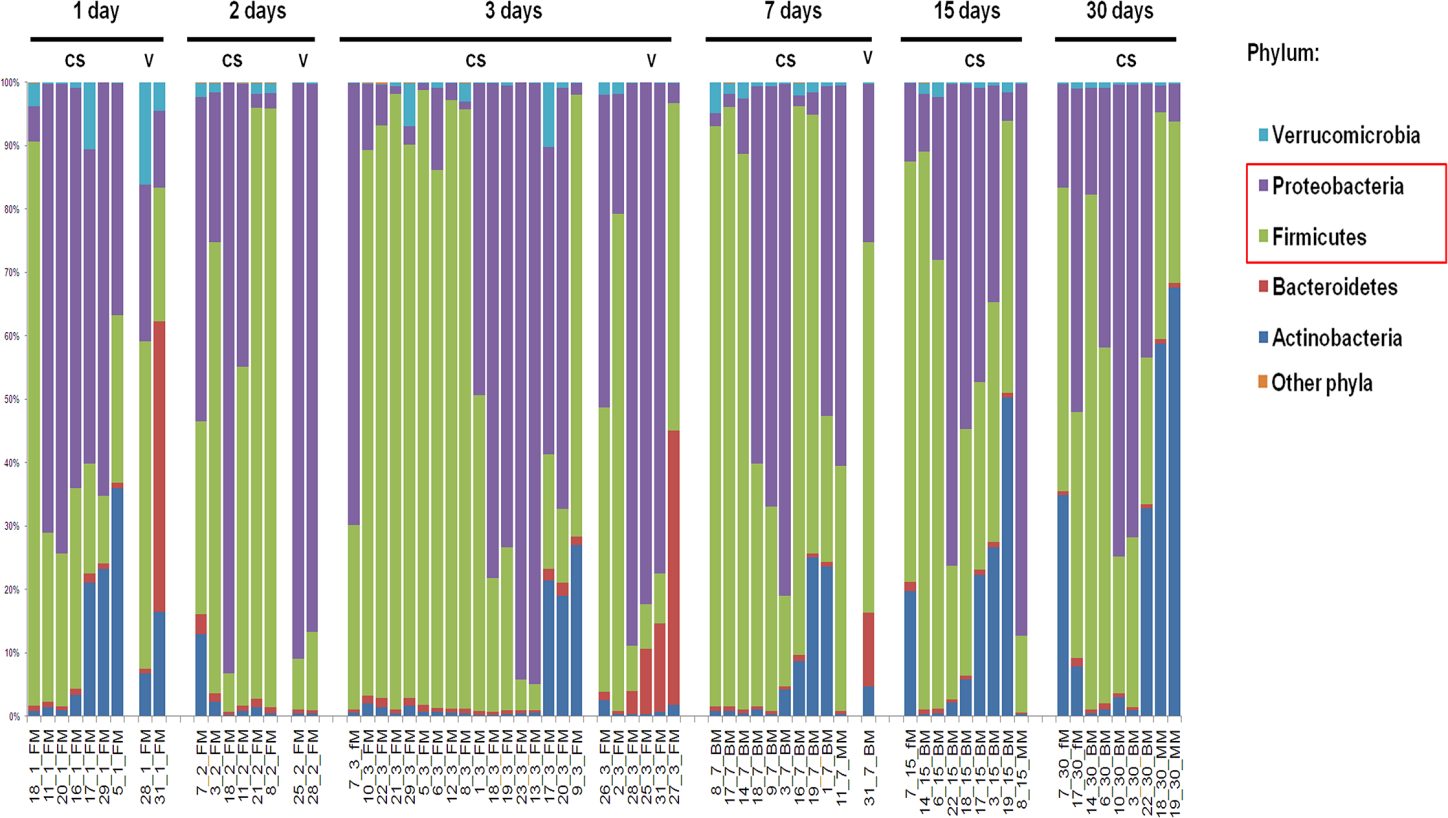
Composition at phylum level of the gut microbiota profiles of 31 infants enrolled in the study for 30 days following birth. At each time point, the gut microbiota was characterized by HITChip-based phylogeny.

**Fig 2:**
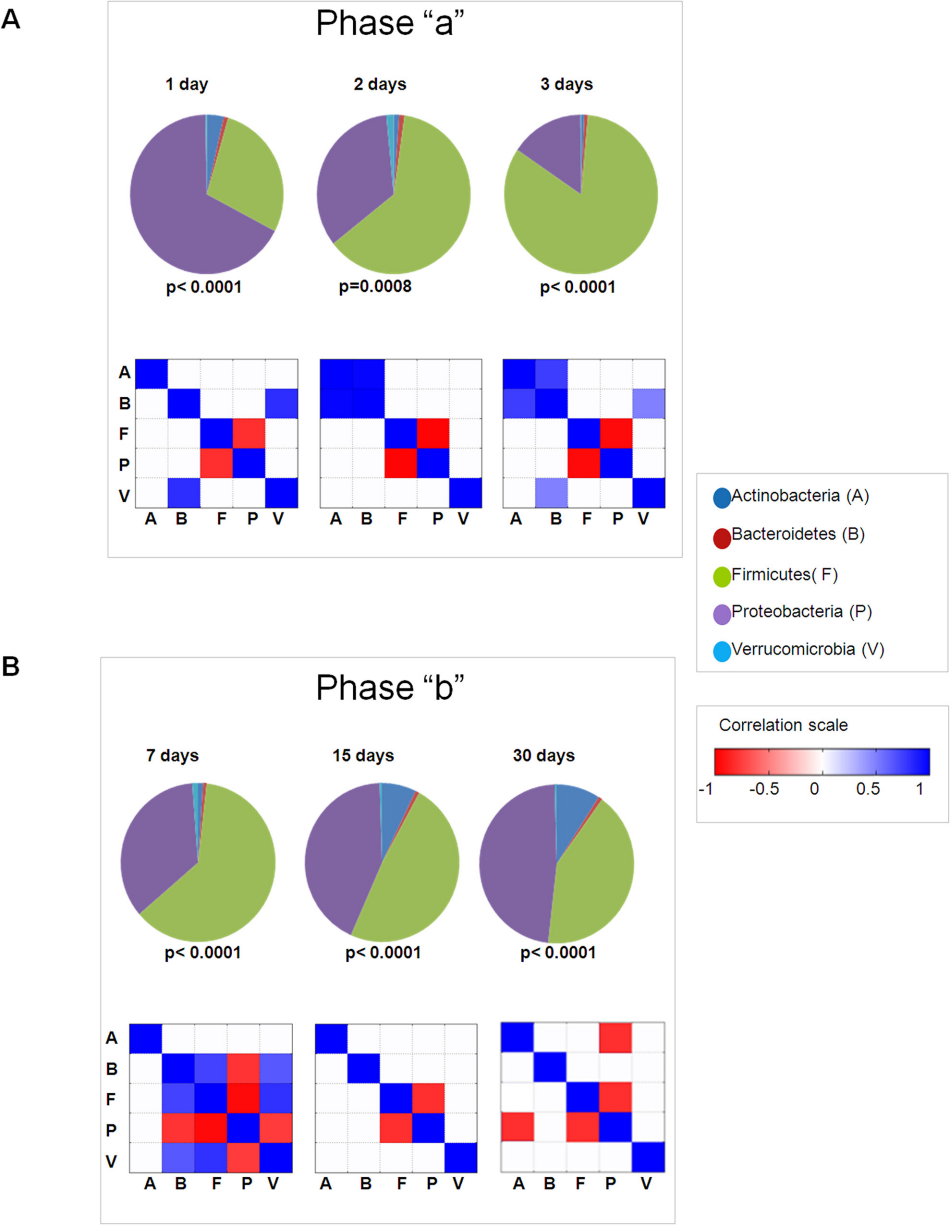
Pie chart representing phyla taxa median values for 30 days following birth. Statistically significant differences in relative abundance at each time point are reported below each graph (Kruskal-Wallis). **Phyla correlation heat maps.** Correlation levels (represented as colored squares) among phyla were calculated by Pearson’s test for all 6 time points. Only squares with a significance *p*<0.05 are shown. Blue squares represent a positive correlation and red squares represent a negative correlation. ***Panel A*** and ***Panel B*** report analyses for 1–3 days (phase “a”) and 7–30 days (phase “b”), respectively.

The temporal evolution of faecal microbiota was determined in a series of 31 healthy newborns, including 25 CS- and 6 V-delivered babies from which relevant metadata were collected (**Table 1; S1 and S2 Tables**). A total of 130 genus-like groups were analysed over 1, 2, 3, 7, 15, and 30 days following birth (**Fig 1; Sheets A-C** in **S3 Table**). The 130 genus-like groups were categorized under 5 phyla, Verrucomicrobia, Proteobacteria, Firmicutes, Bacteroidetes, Actinobacteria, or an “others” phyla group, which included Cyanobacteria (uncultured Chroococcales), Fusobacteria (Fusobacteria), and Spirochaetes (Brachyspira). For phyla with a relative abundance no greater than 0.24% in each analysed sample, no further qualitative and quantitative analyses were considered (**Fig 1; Sheet B in S3 Table**). Proteobacteria and Firmicutes were the major phyla represented at all time points (**Fig 1**). The meconium samples appeared to contain all of the major phyla present in stool during early life and consisted mostly of Proteobacteria and Firmicutes, followed by Actinobacteria, and to a lesser extent by Verrucomicrobia and Bacteroidetes. The progression of microbiota patterns during the first colonization events clearly resembled chaotic series [45]. However, the first colonization could be dependent on pivotal bacterial populations encountered at birth, which conferred a microbiota fingerprint during the first hours and days of life [46,47] (**Fig 1**). Indeed, detectable patterns included: *i*) persistent low levels of Bacteroidetes in CS-delivered babies with postponed colonization after the first days of life, as recently reported [24]; *ii*) high levels of Verrucomicrobia in CS-delivered babies in early days compared to later days; and *iii*) high levels of Bacteroidetes in V delivered babies (**Fig 1**). After grouping samples into two subsets, 1–3 days and 7–30 days, nonparametric Kruskal-Wallis tests were used to identify statistically significant differences among phyla for CS-delivered neonates at each time point (**Fig 2**). When the Shannon diversity indexes, which were calculated at the probe level, for the hybridization patterns for the 130 probes were grouped by feeding, timing, delivery, and gender categories, the only statistically significant difference was between CS and V at 1–3 days (630.16 ± 30.98 SD vs. 653.10 ± 21.46 SD, p<0.03).

**Table 1:** Mother-neonate clinical features and sample numbers and analysis types at each time point.

*Clinical features*										
Gestational age (weeks)								39.00 (37.571–41.571)		
Delivery modality										
Vaginal (%)								6 (19.5%)		
Cesarean section (%)								25 (80.6%)		
*Infant features*										
Infant gender (male:female)								20:11		
Head circumference (cm)								35 (32–37.5)		
BMI (Kg/m^2^)^1^								12.59 (11.6–15.53)		
APGAR^2^								9 (7–10)		
*Sample features*										
Time course	Collected samples							Analyzed by genomics Nr. (%)	Analyzed by metabolomics Nr. (%)	Analyzed by integrated omics Nr. (%)
Time course sampling										
Days	Nr. (%)	FM		BM	fM	MM		9 (13.0%)	17 (24.3%)	7 (15.6)
Day 1	31 (25.2%)	31		0	0	0				
Day 2	30 (24.4%)	30		0	0	0		8 (11.6%)	17 (24.3%)	7 (15.6)
Day 3	28 (22.8%)	28		0	0	0		23 (33.3%)	15 (21.4%)	13 (28.9)
Day 7	15 (12.2%)	0		13	1	1		11 (16.0%)	9 (12.9%)	6 (13.3)
Day 15	10 (8.1%)	0		8	1	1		9 (13.0%)	6 (8.6%)	6 (13.3)
Day 30	9 (7.3%)	0		5	2	2		9 (13.0%)	6 (8.6%)	6 (13.3)
Total	123							69	70	45
Feeding modality^3^										
FM			89 (72.3%)				40 (58.0%)		47 (67.1%)	27 (60%)
BM			26 (21.1%)				22 (31.9%)		16 (22.9%)	14 (31.2%)
fM			4 (3.3%)				3 (4.3%)		5 (7.1%)	2 (4.4%)
MM			4 (3.3%)				4 (5.8%)		2 (2.9%)	2 (4.4%)
Total			123				69		70	45

Data are shown as median and interquartile range or percentage.

^1^BMI, body mass index

^2^APGAR, appearance, pulse, grimace, activity, respiration

^3^FM, first- milk. or colostrum; BM, breast-milk; fM, formula-milk; MM, mixed-milk, BM plus fM.

### Early gut microbiota profiles: strength and direction of correlations among phyla of seminal colonizers

During the first phase of development (1–3 days following birth, phase “a”), CS-associated gut microbiota was prevalently composed of Proteobacteria and Firmicutes. At birth, Proteobacteria (median 63.13) and Firmicutes (median 26.4) were significantly negatively correlated (r = -0.816; p = 0.025). At day 2, the relative abundance of Proteobacteria decreased by nearly half (median 34.1) while the proportion of Firmicutes more than doubled (median 62.3), maintaining a negative correlation (r = -0.982; p = 0.0004). At day 3, the relative abundance of Proteobacteria decreased by almost two-thirds (median 12.94), while Firmicutes increased again (median 69.73), and they remained negatively correlated (r = -0.95; p = 1.08E-09) (**Fig 2A; S4 Table; Sheet A** in **S5 Table**). Indeed, in a culture-based study, it was reported that *Enterobacteria* (*i*.*e*., Proteobacteria) and Gram(+) cocci (*i*.*e*., Firmicutes) were identified as the first gut microbiota colonizers in healthy infants, hence producing a reduced environment favourable for the establishment of later occurring anaerobic bacteria (*Bacteroides* [Bacteroidetes], *Bifidobacterium* [Actinobacteria], and *Clostridium* [Firmicutes]) [22]. In our correlation analysis, during the first phase (phase “a”), Bacteroidetes was positively correlated with Verrucomicrobia at day 1 (r = 0.821; p = 0.023), Actinobacteria at day 2 (r = 0.976; p = 0.0008), and Actinobacteria (r = 0.759; p = 0.0004) and Verrucomicrobia (r = 0.491; p = 0.044) at day 3 (**Fig 2A; S4 Table; Sheet A** in **S5 Table**).

During the second phase (7–30 days following birth, phase “b”), the course of the microbiota composition changed. At day 7, the proportion of Proteobacteria increased again (median 30.40) and Firmicutes decreased proportionally (median 53.91), with the trend continuing at days 15 and 30, when the amounts of Proteobacteria and Firmicutes were similar (median 34.2 *vs*. 38.93, respectively [r = -0.811, p = 0.008] at day 15; median 40.8 *vs*. 35.8, respectively [r = -0.7883, p = 0.048] at day 30) (**Fig 2B; S4 Table; Sheet A** in **S5 Table**).

Starting from day 15, the proportion of Actinobacteria increased until day 30 (median 5.74 and 7.80, respectively). Actinobacteria were most likely of maternal origin, as inferred by the significantly lower *Bifidobacterium* content in colostrum compared to transitional milk microbiota and, in general, lower exposure in CS- compared with V- delivery mode [48]. However, during phase “b”, particularly at day 7, Bacteroidetes was significantly and positively correlated with Firmicutes (r = 0.730; p = 0.017) and Verrucomicrobia (r = 0.822; p = 0.040), which is in agreement with the delayed gut microbiota colonization by Bacteroidetes in CS-delivered babies (**Fig 2B; S4 Table; Sheet A** in **S5 Table**) [24].

**Fig 3:**
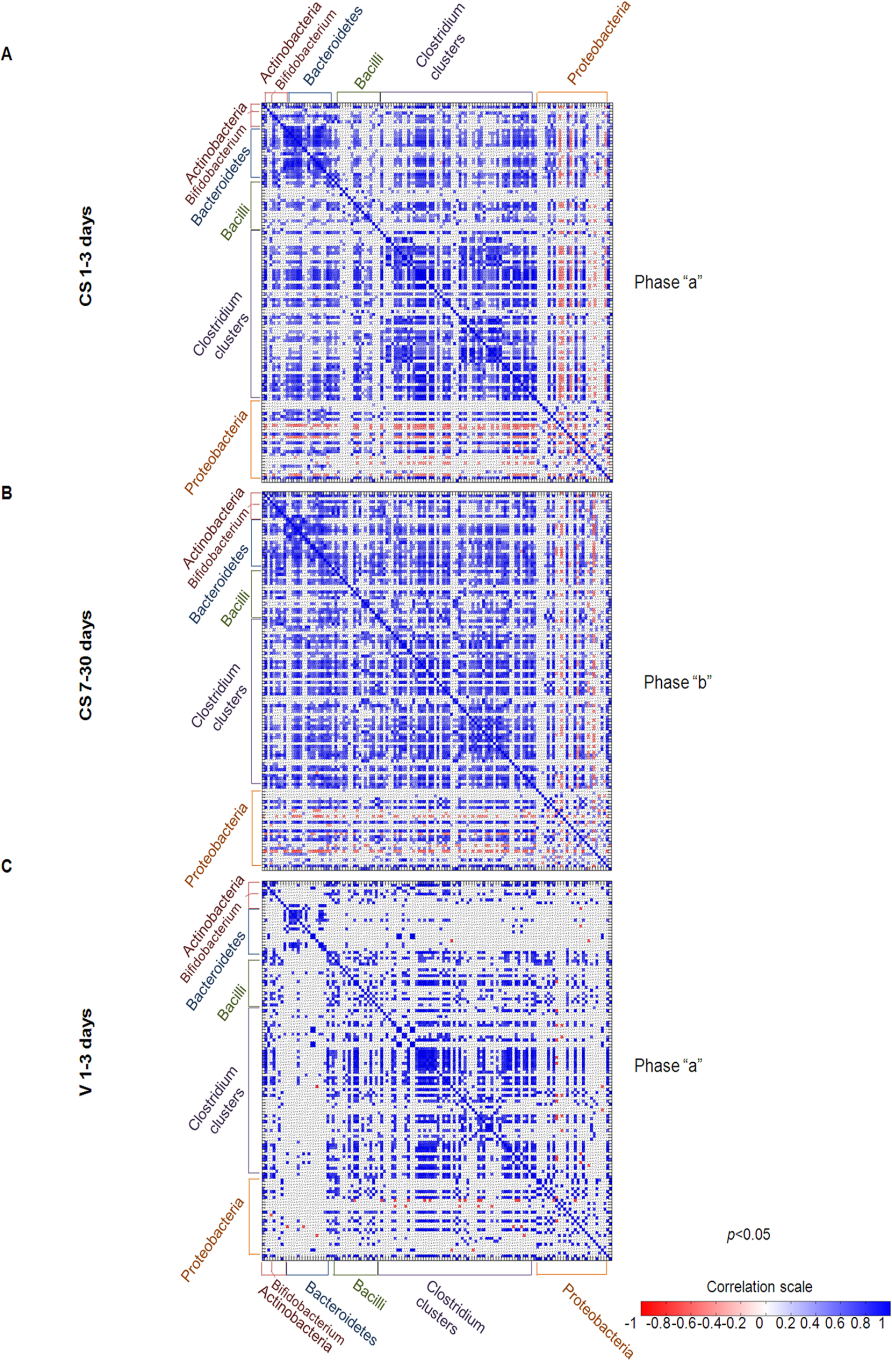
Pearson’s correlation heat maps for the 130 genus-like groups from the HITChip microarray. Panels A, B, and C show the correlation levels (represented by colored squares) among groups, which were calculated by Pearson’s test for all 3 groups (CS-delivered 1–3 days, CS-delivered 7–30 days, and V1-3 delivered days). Only correlations with a significance *p*<0.05 are represented. The colored scale indicates the correlation values.

Based on visual inspection, OTUs associated with the two CS-delivered baby groups at 1–3 and 7–30 days following birth showed similar correlation patterns, although the positive interactions increased from phase “a” to “b” while the negative interactions diminished (**Fig 3, Panels A-B; Sheets B-D** in **S5 Table**). In particular, for the CS-delivered babies at 1–3 days, a moderate positive correlation among Actinobacteria members was observed, with the exception of *Bifidobacterium*, which was significantly correlated (p<0.005) with only 4 OTUs (*i*.*e*., Clostridia, *Clostridium difficile*, *Bacteroides vulgatus*, *Bacteroides uniformis*). Actinobacteria correlation patterns were generally maintained at 7–30 days (**Fig 3, Panel A, Sheet B** in **S5 Table**). During phase “a”, Bacteroidetes members displayed a high general positive correlation. However, the entire group did not correlate or only barely correlated with Bacilli (**Sheet B** in **S5 Table**). Interestingly, Bacteroidetes showed negative or no correlations with Proteobacteria (p<0.005). During phase “b”, among Bacteroidetes OTUs, highly positive correlations were maintained with a general increase in the number of correlations with Bacilli group (p<0.005). Furthermore, positive correlations among *Clostridium* clusters increased throughout phase “b”, while negative correlations with Proteobacteria groups were maintained (p<0.005). During both phases, Bacilli showed a low intra-group positive correlation, which increased over the time course, and a higher correlation with other Firmicutes OTUs but a negative correlation with Proteobacteria (p<0.005). Also, for *Clostridium* clusters, the correlation profile remained constant over the time course. Proteobacteria was the most uncorrelated group, not only with other phyla but also among its own group’s OTUs (**Fig 3, Panel B; Sheet C** in **S5 Table**).

Despite the small number of samples collected from V-delivered babies at 1–3 days (9 stool samples, **S1 Table**), V-associated gut microbiota OTUs correlations were estimated. Upon visual inspection, no or weak correlations were observed compared to CS-groups during phase “a” (p<0.005) (**Fig 3, Panel C**; **Sheet D** in **S5 Table**). The Actinobacteria groups showed a high intra-group correlation and a relatively high correlation with Bacilli and Clostridia clusters but a weak correlation with Bacteroidetes (p<0.005). The Bacteroidetes group showed a high intra-group correlation but a very low or no correlation with other groups (p<0.005). Firmicutes showed high positive intra-group correlations with Actinobacteria and only few with Proteobacteria (p<0.005). Proteobacteria showed no or weakly positive correlations with most groups (p<0.005). For V-delivered babies, the low number of correlations at 1–3 days might be explained by the "inhibition" model proposed by Connell (1977), which suggests that all species resist invasions of competitors [49]. In fact, the first bacterial occupants, originating from the pre-adapted and “ready-to-use” maternal vaginal and faecal microbiota *inoculum*, constitute an already mature and stable niche, pre-empting the space and excluding or inhibiting new pioneers until the former die or are damaged. Only then, later colonizers appear and can reach maturity [50]. It is possible that maternal niches transmitted to the baby during V delivery include already stable and resilient bacteria [50]. However, in CS-delivered neonates, “starter” bacteria belong to different ecosystems such as skin or the external host environment [2]. Moreover, the remarkable increase of CS gut microbiota correlations during “phase b” (**Fig 3, Panel B; Sheet C** in **S5 Table**) suggests two major colonization phases, possibly induced by the passage to transitional or mature breast milk (BM) feeding from day 7 onwards. The first colonizers, generally opportunistic species (*i*.*e*., *Pseudomonas aeruginosa*, *Staphylococcus*) are able to occupy “empty” environments, triggering different mechanisms of subsequent species settlement. During phase “a” the richness, diversity, and complexity of interactions among OTUs are low but immediately tend to increase over time, indicating that the relatively simple and scarce bacterial communities present in the first week can tolerate the incoming establishment of new species or pre-existing pioneers at lower growth kinetics [27,51–54]. However, after “setting” the new environment, colonization by new species can be facilitated, producing and enhancing positive correlations [50]. In a few cases, pioneers inhibit further species until a release of resources through negative correlations. During phase “b” the number of interactions among OTUs may increase proportionally with the higher intake of nutrients, linked not only to BM composition but also to the increased volume of milk intake [55,56]. It has long been known that the concentration of many nutrients, such as carbohydrates (*i*.*e*., lactose), lipids (*i*.*e*., fats), and bioactive compounds (*i*.*e*., minerals, proteins, water-soluble vitamins), increases from FM to BM, while growth factors, antimicrobial factors, IgA, and lymphocytes decline [55].

### Co-occurrence networks of OTUs

**Fig 4:**
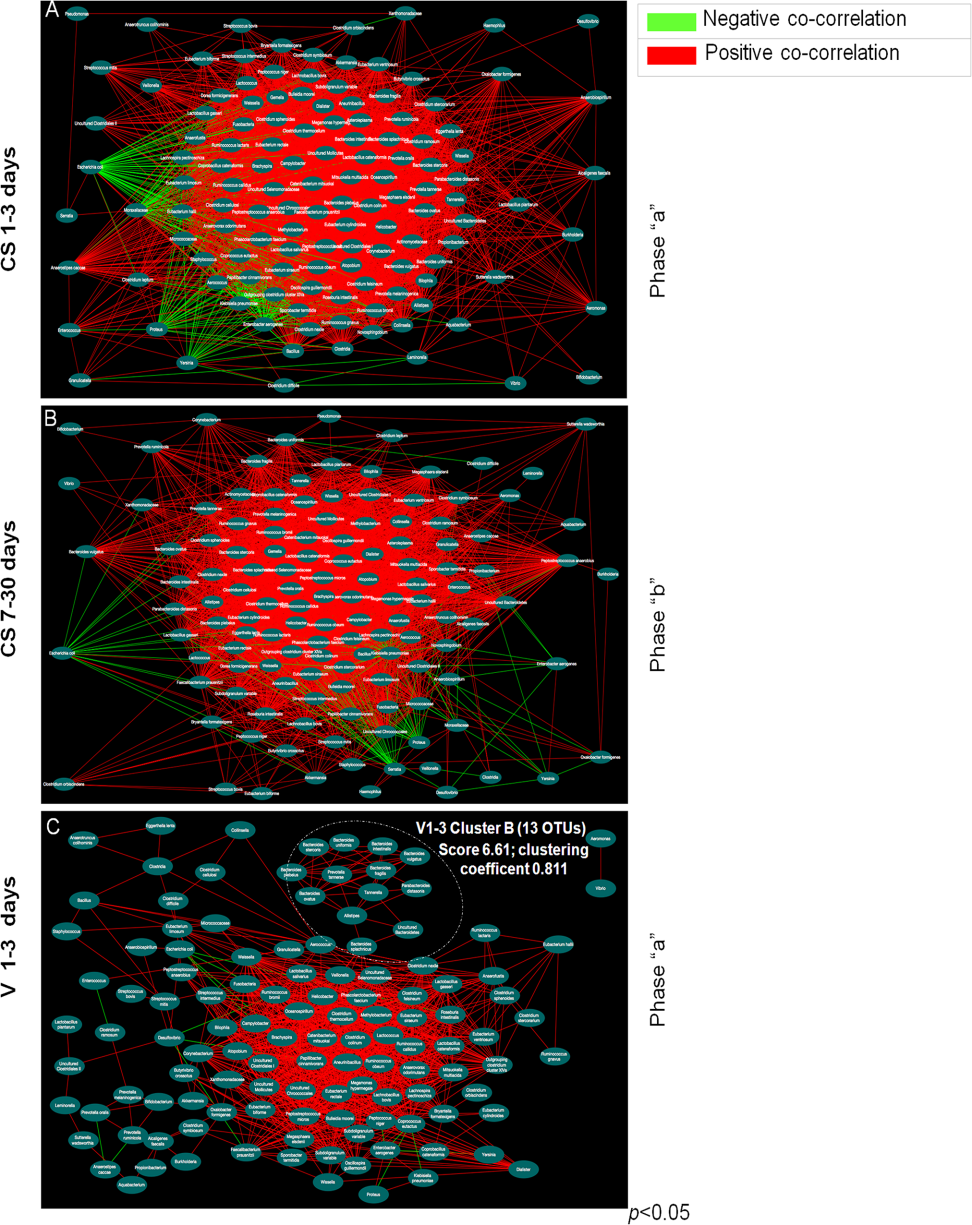
Graphical representation of OTUs co-occurrence networks. **Panel A** shows the OTUs co-occurrence network for CS-delivered babies at 1–3 days following birth (see **sheet B** in **S5 Table** for details). **Panel B** shows OTUs co-occurrence network for CS-delivered babies at 7–30 days following birth (see **sheet C** in **S5 Table** for details). **Panel C** shows OTUs co-occurrence network for V-delivered babies at 1–3 days following birth (see **sheet D** in **S5 Table** for details). Red line indicates a positive correlation and a green line indicates a negative correlation. Pearson’s test was used to evaluate the correlation amongst OTUs (statistical significance was assessed with *p*<0.01).

In order to study the co-occurrence networks of the relative abundance of OTUs, microbial clusters were visualized for CS- and V-delivered babies at 1–3 days (**Fig 4, Panels A, C**) and CS-delivered babies at 7–30 days (**Fig 4, Panel B**). The central portion of each panel shows the gut microbiota “core”, represented by the network of OTUs with the highest clustering coefficient, a function related to the number of correlations that in most instances was positive. In **Panel A**, within the 130 OTUs framework, 124 positive and 53 negative correlations were found. In **Panel B,** 121 correlations were positive and 42 negative. In **Panel C,** 103 correlations were positive and 13 negative, confirming a reduced number of interactions in V-delivered compared to CS-delivered babies at days 1–3. One main cluster was present in CS-delivered babies at both 1–3 and 7–30 days, composed of 55 and 62 OTUs, respectively, while two clusters were identified in the V-delivered babies, composed of 33 (*i*.*e*., *cluster A*) and 13 OTUs (*i*.*e*., Bacteroidetes *cluster B*) (**Sheet E** in **S5 Table**). In order to assess the uniqueness of OTUs (single *network-tailored structure*) or, alternatively, commonness (*network “core” structure*), co-occurrence clusters were interpreted with reference to presence/absence in the CS 1–3, CS 7–30, and V 1–3 groups. The following gut microbiota network structures were identified: *i*) unique OTUs associated with the CS-delivered group at 1–3 days; *ii*) unique OTUs associated with the CS-delivered group at 7–30 days; *iii*) unique OTUs associated with the V-delivered group at 1–3 days; i*v*) OTUs shared by groups *i* and *iii*; *v*) OTUs shared by groups *i* and *ii*; *vi*) OTUs shared by all groups (**S1 Fig; Sheet F** in **S5 Table**). The “core” structure (*i*.*e*., *structure vi*) was composed of 30 stable microbiota (SM) OTUs belonging to: Actinobacteria (1), Bacteroidetes (4), Cyanobacteria (1), Firmicutes (19), Proteobacteria (4), and Spirochaetes (1) (**Sheet F** in **S5 Table**). The identification of a defined core, independently associated with the delivery mode and lactation stage, provides support for the Savage theory, which is based on the assembly of gut microbiota as a “niche-driven process” that results in the establishment of a SM, followed by variable microbiota (VM) [57].

In the Savage model, specific niches, which are characterized by nutrients, habitat, and evolutionary competition, select the species and their abundance and distribution, driving the diversity of gut microbiota. The SM members are ‘autochthonous’ and are always found in individuals regardless external stimuli, which drives the onset of delivery mode- and feeding-dependent specific niches, likely shaped by allochthonous microbes [58]. This theory may explain some ecological features of gut microbiota, including progression in the early stages of microbiota onset and the evolution of neutral and niche distributions of microbial inhabitants (**S1 Fig)** [59–64].

### NMR metabolic profiling

Carr-Purcell-Meiboom-Gill (CPMG)-NMR spectra were obtained from 70 faecal samples from 21 newborns collected at 1, 2, 3, 7, 15, 30 days after delivery (**Table 1; S1 Table**). Thirty metabolites were identified and quantified and three remained unidentified, reported as U1, U2, and U3 (**S6 Table**). NMR spectra were characterized by resonances associated with short-chain fatty acids (SCFAs), choline and derived metabolites (*i*.*e*., dimethylamine, trimethylamine), alcohols, acetoin, amino acids (AAs) and derivatives (*i*.*e*., 3-hydroxyisovalerate), tricarboxylic acid cycle intermediates and products of energy metabolism (*i*.*e*., lactate, succinate, creatinine), formate, and sugars (fucose) and N-acetyl derivatives, including those originating from both diet (*i*.*e*., HMOSs containing N-acetylglucosamine residue) and gut mucosa, all of which were derived from microbial saccharolytic and proteolitic fermentation and gut mucin degradation [36] (**S6 Table**). It has previously been shown that HMOSs from colostrum (cHMOSs) can modulate the signalling pathways of immature mucosa, associated with IS, which hyper-reacts to newly colonizing unfamiliar bacteria [65].

#### Time-dependence of NMR metabolic settlement

A comparison of the ^1^H-NMR spectral profiles of samples collected at 1, 2, 3, 7, 15, and 30 days showed that samples collected within the first 3 days of life had fewer metabolites compared to samples obtained at days 7, 15, and 30 (**S2 Fig; S7 Table**). This rise in metabolites (*e*.*g*., ^1^H-NMR profiles of newborn no. 18, **S1 Table; S2 Fig**) likely reflects the increasing transit of milk low molecular weight (LMW) components from the small intestine to the colon and the progressive gain of gut microbial biomass [66,67]. In particular, several physiological factors can explain these metabolic changes during days 7 to 30, including: *i*) increased breast milk intake volume; *ii*) increased intake of nutrients related to milk maturation; and *iii*) decreased small intestine assimilation of LMW nutrients, related to the gut closure process [68]. Interestingly, in humans the gut closure process is completed between 3 and 7 days after delivery [68]. Our results show that the total level of metabolites increases over time, reflecting higher gut microbiota metabolic activity, possibly related to breast milk maturation and progressive intake.

#### Time- dependence of NMR metabolic profiling

**Fig 5:**
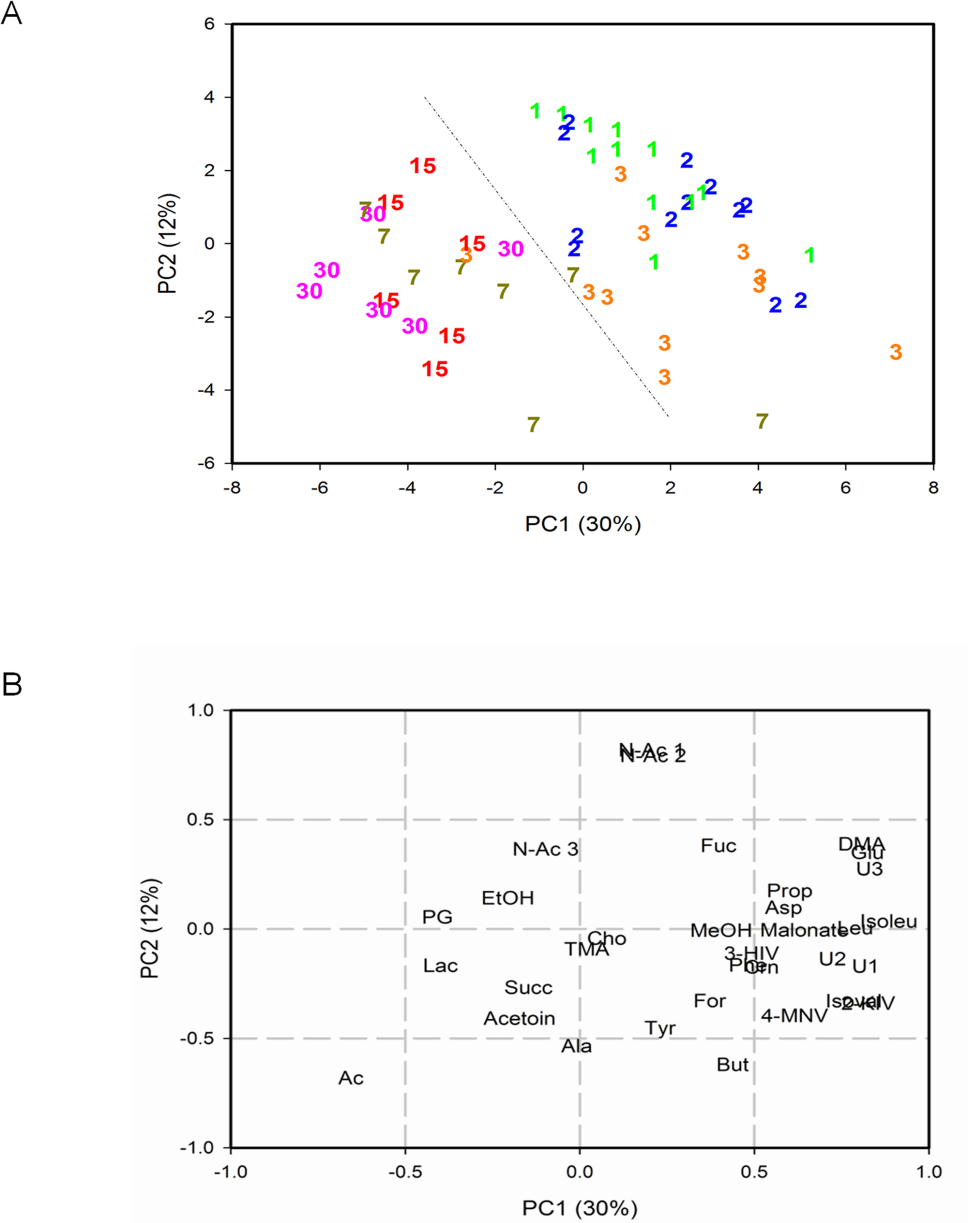
Principal Components Analysis (PCA) of metabolic profiles from 55 CS-delivered newborns. Samples were analysed over the 30 days following birth. **Panel A**: PC score plot. **Panel B**: loading plot. The first two components explained 42% of the total variance.

A PCA analysis was performed to evaluate time-dependent changes in 55 metabolic profiles from 16 CS-delivered newborns. The first two components explained 42% of the total variance (30% and 12% for PC1 and PC2, respectively). The score plot showed a net separation along PC1 between faecal samples collected at 1, 2, and 3 days and 7, 15, and 30 days (**Fig 5, Panel A**). In particular, considering the scores at high PC1 values, the samples from 1–2 days clustered along PC2 more so than day 3 samples (**Fig 5, S3 Fig**). The loading plot showed that, along PC1, faecal samples at days 1, 2, and 3 had higher levels of isoleucine, leucine, glutamate, aspartate, dimethylamine, isocaproic acid, propionate, 2-hydroxy-3-methylbutyrate, isovalerate, U1, and U2 than those collected at 7, 15, and 30 days (**Fig 5, Panel B; S7 Table**). In contrast, samples from 3, 7, 15, and 30 days were characterized by higher acetate levels compared to those collected within the first two days (**S7 Table**). Moreover, the majority of faecal samples at days 1 and 2 had higher N-acetyl-derivative (NAc1 and NAc2) levels than those collected at days 3, 7, 15, and 30, while NAc3 remained unchanged (**S8 Table**; **Fig 5, Panel B**). At day 1, NAc may originate from the N-acetylated glycoproteins of gut mucosa, while at day 2 NAc may be contributed by ingested milk components, such as N-acetylglucosamine-containing oligosaccharides or glycoproteins [69]. NAc and acetate can be found at days 1–30, suggesting a substrate/product relationship that changes as a function of time and is dependent on changes in gut microbiota. The level of butyrate was lower at day 1 than at day 3 (**Fig 5, Panel B),** remained steady until day 7, and then declined by half between days 15 to 30, following the concentration of milk carbohydrates (**S8 Table)** [70].

The metabolite content in the first 3 days of life is characterized by high AAs and AA catabolites and low acetate levels, while at days 15 and 30 the levels are reversed, suggesting an inverse relationship between protein/AA and acetogenic metabolism (**S8 Table**). For instance, 2-hydroxy-3-methylbutyrate and isovalerate originate from branched chain AAs (BCFAs) [71] and are generally present in low quantities compared to SCFAs in the large intestine and originate exclusively from protein breakdown, thus representing illustrative pathway markers [72]. Moreover, milk and endogenous luminal proteins are consumed by gut microbiota metabolism and by exocrine pancreatic proteases activity, releasing peptides, oligopeptides, and AA [73]. In accordance with colon anatomy and physiology, putrefactive protein processes become quantitatively more important in the distal bowel, where the luminal pH is nearer to the optimal for protease activity [74]. AAs can be directly incorporated into bacterial cells as building blocks of proteins or metabolized by the microbiota, producing a complex mixture of metabolic end products including SCFAs, BCFAs, and H_2_S [75]. In contrast, microbial production of acetate originates from carbohydrates, oligosaccharides, or AAs depending on environmental conditions and microbiota species [76]. Acetate makes up around 60–75% of the total faecal SCFAs being formed by many habiting colon bacteria, with approximately one-third coming from reductive acetogenesis, the process through which acetate is produced from an electron acceptor (*i*.*e*., CO_2_) and electron donors (*e*.*g*., H_2_, CO, formate, etc.) by anaerobic bacteria (acetogens, methanogens, and acetate-producing bacteria such as *Clostridium* spp.) [77] via the reductive acetyl-CoA, or Wood-Ljungdahl, pathway [76,78]. In this pathway, CO_2_ is reduced to CO, which is then converted to acetyl-coenzyme A through CO dehydrogenase and acetyl-CoA synthase. The former catalyzes the reduction of CO_2_ and the latter combines the resulting CO with a methyl group to give acetyl-CoA [78,79].

The higher levels of AAs and their catabolites observed in the first 3 days after delivery compared to days 7, 15, and 30 suggest a shift in colon microbial fermentation consistent with milk composition changes during the lactation process. Indeed, it is well known that FM contains higher levels of proteins and lower levels of lactose and fatty acids than BM [55,56].

### Correlations between metabolite-metabolite and metabolite-OTUs

To investigate metabolic activities associated with OTUs during the first month of life, the relationships among metabolites and between metabolites and OTUs were evaluated by Spearman’s rank-order correlation on sample sets analysed by both by ^1^H-NMR and HITChip microarray (**S1 Table**). In particular, the correlations between OTUs and metabolites were focused on microbial metabolism associated with acetate and AAs and their degradation intermediates (*e*.*g*., dimethylamine, and NAc1, NAc2). There were a significantly inverse correlations between acetate and isoleucine, leucine, 2-hydroxy-3-methylbutyrate, isovalerate, dimethylamine, glutamate, aspartate, propionate, NAc1, and NAc2 (**Sheet A** in **S9 Table,**). Such inverse correlations, highlighted by PCA, reflect the metabolic changes observed at days 1–3 and 7–30.

**Fig 6:**
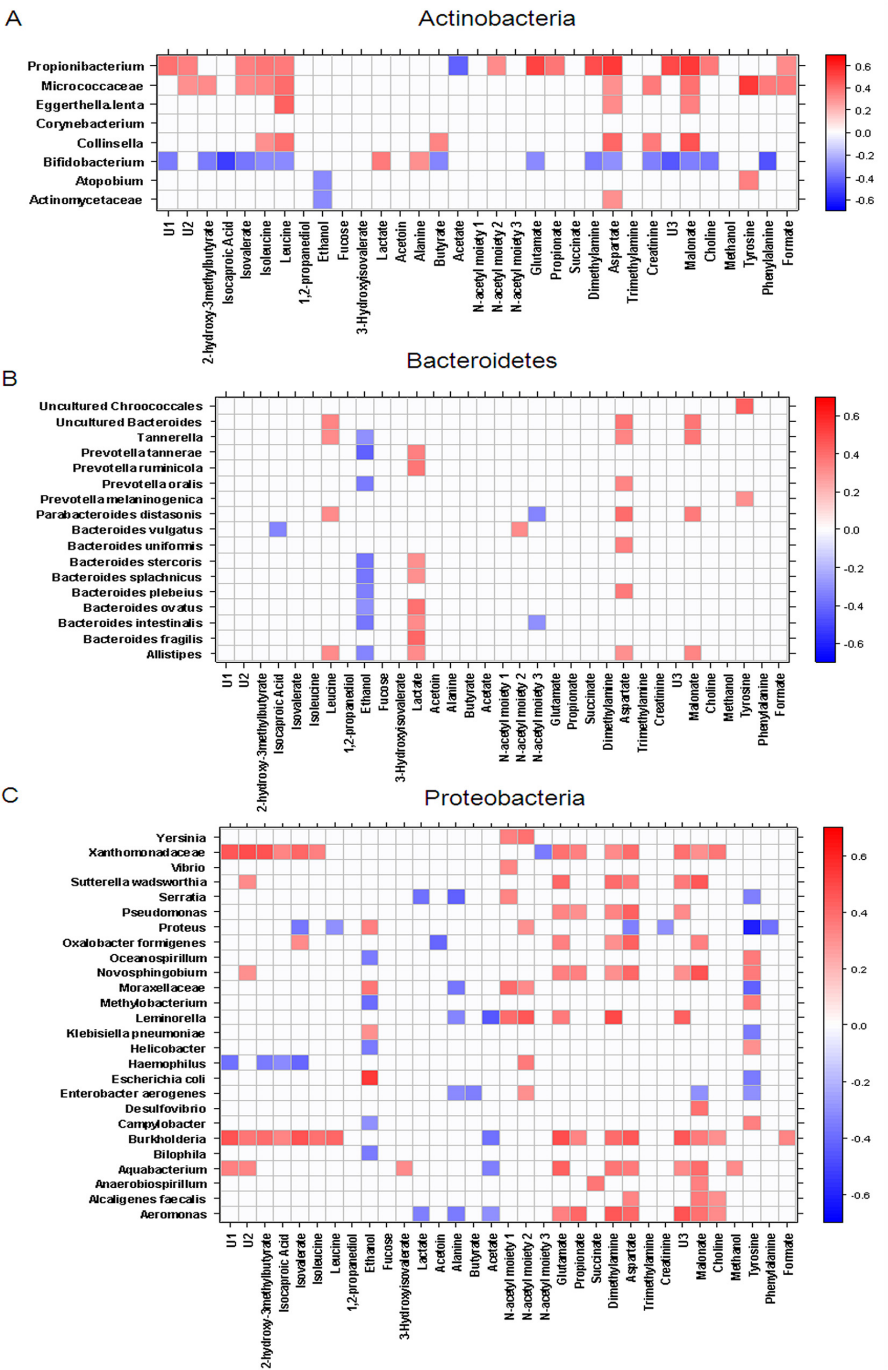
Correlation heat-map between OTUs and faecal metabolites. Significant correlations (p<0.05) for Actinobacteria (**Panel A**), Bacteroidetes (**Panel B**), Proteobacteria (**Panel C**). 2OH3MB: 2-hydroxy-3-methylbutyrate; Isocapr: Isocaproate; Isoval: Isovalerate; Ile: Isoleucine; Leu: Leucine; EtOH: Ethanol; Fuc: Fucose; 3OH-Isoval: 3-hydroxyisovalerate; Lac: Lactate; Ala: Alanine; But: Butyrate; Ac: Acetate; N-Ac: N-Acetyl moiety; Glu: Glutamate; Suc: Succinate; DMA: Dimethylamine; Asp: Aspartate; TMA: Trimethylamine; Cr: Creatine; MA: Malonate; Cho: choline; MeOH: Methanol; Tyr: Tyrosine; Phe: Phenylalanine; For: Formate.

**Fig 7:**
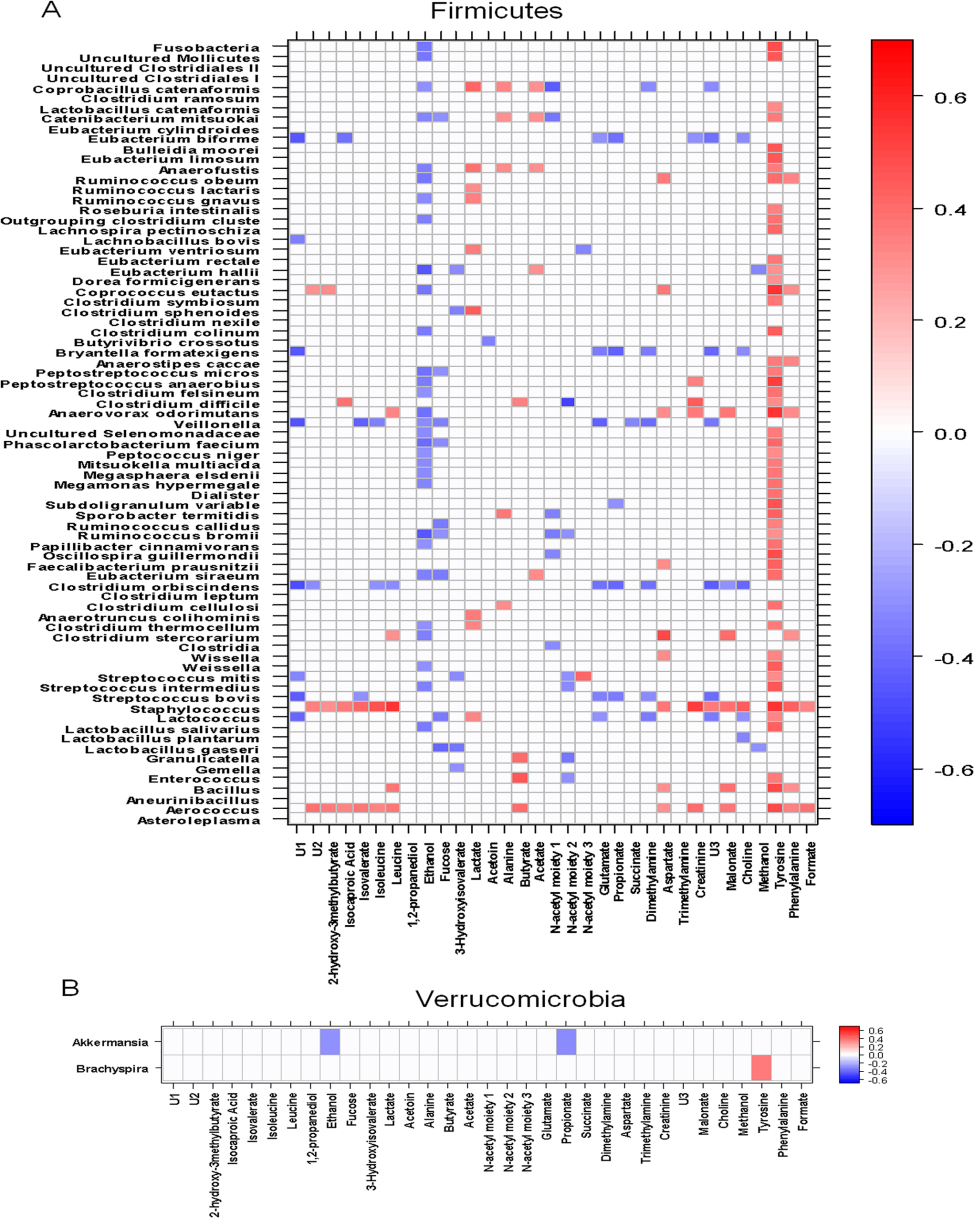
Correlation heat-map between OTUs and faecal metabolites. Significant correlations (p<0.05) for Firmicutes **(Panel A)** and Verrucomicrobia **(Panel B)**. 2OH3MB: 2-hydroxy-3-methylbutyrate; Isocapr: Isocaproate; Isoval: Isovalerate; Ile: Isoleucine; Leu: Leucine; EtOH: Ethanol; Fuc: Fucose; 3OH-Isoval: 3-hydroxyisovalerate; Lac: Lactate; Ala: Alanine; But: Butyrate; Ac: Acetate; N-Ac: N-Acetyl moiety; Glu: Glutamate; Suc: Succinate; DMA: Dimethylamine; Asp: Aspartate; TMA: Trimethylamine; Cr: Creatine; MA: Malonate; Cho: choline; MeOH: Methanol; Tyr: Tyrosine; Phe: Phenylalanine; For: Formate.

Correlations between OTUs and acetate, AAs, NAc1, and NAc2 were also examined, with a focus on OTUs that were positively correlated with AAs and their derivatives and inversely correlated with acetate. Among Actinobacteria OTUs, *Propionibacterium* was positively correlated with leucine, isoleucine, isovalerate, propionate, glutamate, aspartate, and dimethylamine and negatively correlated with acetate (**Fig 6, Panel A**). Similarly, among Proteobacteria, *Burkholderia* was positively correlated with leucine, isoleucine, isovalerate, propionate, glutamate, aspartate, dimethylamine, and malonate and negatively correlated with acetate. Other bacterial OTUs were positively correlated with only AAs and their derivatives. In particular, Xanthomonadaceae (Proteobacteria) and *Staphylococcus (*Firmicutes) were positively correlated with isoleucine, 2-hydroxy-3-methylbutyrate, isocaproate, isovalerate, and aspartate (**Fig 6, Panel C; Fig 7, Panel A**). Other Proteobacteria, such as *Enterobacter aerogenes*, *Leminorella*, and *Yersinia*, were positively correlated with NAc1 and NAc2 and negatively correlated with butyrate (*E*. *aerogenes* only) or acetate (*Leminorella* only) (**Fig 6, Panel C**). Among Actinobacteria, *Bifidobacterium* was negatively correlated with leucine, isoleucine, 2-hydroxy-3-methylbutyrate, isocaproate, and isovalerate as well as with glutamate, malonate, and dimethylamine (**Fig 6, Panel A**). Among Firmicutes, *Clostridium* OTUs were positively correlated with acetate levels (**Fig 7, panel A**).

## Conclusions

In conclusion, gut microbiota phylogenetic tracking in meconium/faecal samples collected in the first 30 days of life from CS-delivered babies revealed a largely chaotic development, within which Proteobacteria and Firmicutes were the main phyla represented. Low levels of Bacteroidetes and Verrucomicrobia were present during the first 30 days and Actinobacteria starting at day 15. In the first 3 days of life, CS-associated gut microbiota was mostly composed of Proteobacteria and Firmicutes, which were inversely correlated, confirming that the first colonizers in infants create a reduced environment favourable for the establishment of further niches of anaerobic bacteria occurring later in life. Between 7 and 30 days, the trend in microbiota composition started to invert and there was an increase in maternal-originating Actinobacteria from days 15 to 30, when milk composition and intake increased availability of nutrients as well as *Bifidobacterium*. The microbial correlation analysis highlighted that, during the first colonization phase (1–3 days), the diversity and complexity of interactions among OTUs were already “structured” in groups of positive and negative interaction niches. During the second phase (7–30 days) interactions among OTUs increased considerably, likely the result of increased milk nutrient intake favouring bacterial activities.

No or weak correlations were found in V-delivered babies, whose representative bacterial group mainly consisted in Bacteroidetes, possibly due to the first maternal-driven bacterial inhabitants slowing subsequent bacterial colonization by ecological exclusion or inhibition. The identification of a defined core, independent from delivery mode and feeding stage, supports the “niche-driven process” model that results in the establishment of a stable community assembly in gut microbiota (SM), the precursor of VM.

NMR–based metabolic profiling was used for the first time to investigate faecal metabolome in at-term newborns soon after birth. This analysis highlighted seminal relationships between faecal microbiota development and metabolic pathways. PCA results and correlations among metabolites and OTUs suggest that the faecal metabolome is characterized by OTUs with highly specialized metabolic functions are triggered by milk nutrient digestion, microbial colonization, and host physiology. In the first two days the gut microbial biomass metabolism was lower compared to subsequent days, as shown by ^1^H-NMR spectra. Metabolic profiles reveals higher protein metabolism, with high levels of free AAs and BCFAs and low levels of acetate and butyrate associated with high levels of some Proteobacteria families and low levels of Bifidobacteria and Clostridiaceae. In contrast, high levels of acetogenic metabolism were observed from days 3 to 30 and were correlated with increased levels of some *Clostridium* families, which metabolize carbohydrates to acetate and SCFA.

**Fig 8:**
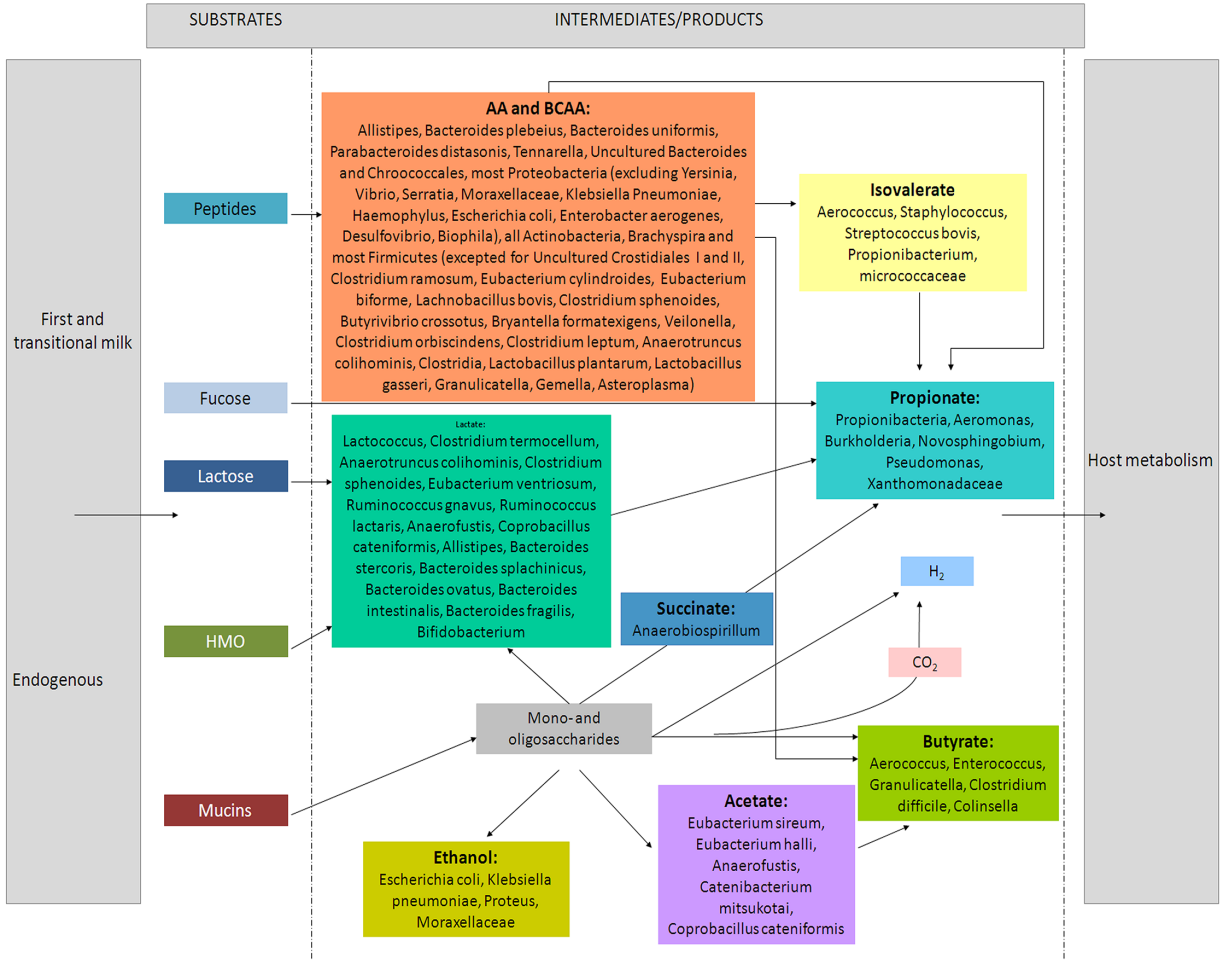
Model of microbial metabolism in perinatal babies under trophic pathways associated with transitional/mature milk. The integration of genomic and metabolomic pipelines suggests a functional picture of microbial metabolism, primed by transitional/mature milk trophic pathways, during early development.

Finally, the integration of genomic and metabolomic pipelines has allowed us to suggest a functional picture of metabolism by microbial OTUs, primed by transitional/mature milk trophic pathways, during perinatal development (**Fig 8**).

## Materials and Methods

### Study design and enrollment of subjects

This study is part of the Italian Baby Trial (IBT), under a collaborative project between Wageningen University (Wageningen, The Netherlands) and Children’s Hospital and Research Institute OPBG (Rome, Italy). Mother and neonate pairs were recruited at the Department of Obstetrics and Gynaecology, San Camillo Hospital (Rome, Italy). Thirty-one healthy mothers and their caesarean (CS) or vaginally (V) delivered neonates were included in the study after careful evaluation for exclusion criteria (*e*.*g*., pre-term delivery, newborn GI and immunological disorders, antibiotic administration during pregnancy) (**Table 1; S1 Table** and **S2 Table**).

In the first study phase (1–3 days following birth), mother/newborn clinical data were collected during hospitalization (*e*.*g*., gestational age, head circumference, BMI [body mass index], APGAR scores [appearance, pulse, grimace, activity, respiration indexes]), while domicile mother’s interviews were performed during the entire second study phase (7–30 days following birth).

At fixed time-points (*e*.*g*., 1, 2, 3, 7, 15 and 30 days), data on baby feeding modality were recorded from 121 nurses’ and mothers’ interviews. Newborn stools were collected during hospitalization (days 1–3) and at home (days 7–30). Feeding information included: *i*) colostrum or first milk (FM) at 1–3 days; *ii*) transitional and mature breast milk (BM) after 3 days; *iii*) formula (fM) or mixed milk (MM, *i*.*e*., BM plus fM) at 7–30 days (**Table 1; S1 Table**).

### Ethics statement

The study protocols were approved by the Ethics Committee of the San Camillo-Forlanini Hospital (protocol 460/CE; 27/03/2012) and by the Institutional Review Board of the Bambino Gesù Children’s Hospital (protocol 295 LB; 16/05/2012). Informed written consent was obtained from all mothers-to-be on behalf of themselves and their neonates.

### Stool collection and sample barcoding

Fresh neonatal faeces were collected from diapers with a sterile gauze inlay to prevent liquid absorption and were transferred into a stool collection container (FL Medical, Italy) at 4°C. Samples were stored at -80°C prior to DNA and metabolite extractions for HITChip and metabolome analyses, respectively (**S1 Table**).

### DNA extraction

Genomic DNA was extracted from faecal samples and subjected to extraction quality control (QC) as described previously with slight modification [80]. Briefly, to overcome the tarry texture of meconium, 1- and 2-day stool samples were resuspended and homogenized in 1.5 ml PBS. In order to guarantee the representativeness of bacterial DNA (*i*.*e*., Gram(-), Gram(+), acid-alcohol-fast bacteria), the homogenate meconium and faecal samples were submitted to mechanical and thermal lysis. After centrifugation at 52×g for 5 min, the supernatant was subjected to NucliSens easyMAG automatic DNA extraction, according to the manufacturer’s instructions (BioMerieux, France). Alternatively, for meconium samples, DNA was extracted using the QIAamp DNA stool mini kit (Qiagen, Germany). Finally, DNA was eluted into 100 μl TE buffer, quantified using a NanoDrop ND-1000 spectrophotometer (Thermo Scientific, USA), and adjusted to a concentration of 10 ng/μl for use as a template for microarray hybridization and analysis. Gut microbial taxa were analysed using the phylogenetic microarray HITChip [81]. Phylogenetic microarrays were custom-synthesized by Agilent Technologies (Agilent Technologies, Palo Alto, CA) and hybridized with DNA samples that successfully passed the QC for HITChip hybridization reproducibility [81,82], (**Table 1**, **S1 Table**).

### HITChip microarray data extraction and analysis

Data were extracted from microarray images using the Agilent Feature Extraction software, version 9.1 (http://www.agilent.com), and analysed in terms of normalization and hybridization reproducibility as previously described for faecal samples from adults [81,83]. The intensity value of each probe, associated with a specific microbial taxon [81], was normalized using quantile normalization applied to the collective spatially normalized measurements of each sample [82] (**Table 1; Sheet A in S3 Table**). In detail, samples were hybridized in duplicate and a >0.95 Pearson correlation coefficient of the natural logarithm of spatially normalized signals, excluding outlier spots [82], was considered indicative of satisfactory HITChip hybridization reproducibility. Robust probabilistic averaging (RPA) was used to calculate relative abundance. The accuracy of each probe was determined and the less accurate probes were weighted lower in the summarization [84]. HITChip microarray data, expressed as normalized hybridization signals, are provided as part of the supporting information in **Sheet A in S3 Table** (A_ Original HITChip data), consistent with PLOS data policy.

### Statistics and network analyses

Measures of hybridization data obtained from 130 HITChip genus-like groups were computed as medians with interquartile ranges (IQR) for each time point (**S4 Table**) and the corresponding 130 OTUs were grouped at phyla (**Fig 1**) and bacterial family levels (*data not shown*). Shannon diversity index (including richness and evenness) was calculated at the probe level in R, and groups (feeding, timing groups, delivery, gender) were compared by Student’s T test. Intra-group and inter-group correlations at each time point among phyla relative abundance values were evaluated by Kruskal-Wallis tests (**Fig 2**). In all analyses, statistical significance was defined as *p*<0.05. Correlation matrices were obtained by Pearson coefficient and plotted as heat maps for each time point (**Fig 1**) and for CS1-3, CS7-30, and V1-3 data subsets (**Fig 2; Sheets B-D in S5 Table**). Co-occurrence network analyses of relative OTUs abundance in V and CS babies were focused on CS1-3, CS7-30, and V1-3 groups (**Sheet E in S5 Table**). Graphical representations of CS1-3, CS7-30, and V1-3 co-occurrence OTUs networks for values of *p*<0.05 (**Fig 4**) were visualized with Cytoscape 2.8.2 version and the presence of main microbial clusters was evaluated using an MCODE plug-in. Only clusters with the highest score and clustering coefficient were calculated with the Network Analyzer plug-in of Cytoscape 2.8.2 (**S1 Fig; Sheet F in S5 Table** *)*. Additional statistical analyses were performed with SPSS (version 20, IBM Corporation, Armonk, NY, USA), GraphPad Prism (GraphPad Software, version 5.01), and R (version 3.0.3). V groups at 1 and 2 days were excluded from the statistical analyses but summarized qualitatively.

### NMR-based metabolomic analysis

#### Metabolite extraction for 1H-NMR analysis

A subset of faecal samples (**Table 1**, **S1 Table**) was suitable for metabolite extraction (weight >100 mg) and further analysed by ^1^H-NMR spectroscopy (**Table 1**and **S1 Table)**. Faecal water was obtained as previously described with the following modifications [85,86]. Briefly, frozen faeces were weighed and homogenized by vortexing into cold PBS/D_2_O (1.2 ml). After centrifugation at 10,000×g for 10 min at 4°C, supernatants were filtered through a cell strainer (40 μm pore size) and further centrifuged. Aliquots of 500 μl supernatant (*faecal water*) were added to 500 μl of D_2_O containing 2 mM trimethylsilyl-sodium-deuterated propionate (TSP) as an internal standard (IS), and 0.05% sodium azide. Samples were stored at -80°C until ^1^H-NMR analysis.

#### ^1^H-NMR spectra acquisition

^1^H-NMR analysis was performed at 298 K using a Bruker Avance 400 spectrometer (Bruker BioSpin, Germany) equipped with a magnet operating at 9.4 Tesla and at 400.13 MHz for ^1^H frequency. The standard pre-saturation sequence used for this study, a Carr-Purcell-Meiboom-Gill (CPMG) sequence, prevented the presence of signals from larger, slower moving molecules (*e*.*g*., polypeptides or polysaccharides) and kept the narrow signals from low molecular weight molecules. A set of preliminary experiments was carried out to avoid any unwanted T_2_ effect. The relaxation delay was 6.55 s, during which a low-power selective pulse was applied for solvent signal pre-saturation, and 128 scans of 64 K points each were collected, preceded by 4 dummy scans. CPMG sequence consisted of 256 spin-echo cycles with an echo time of 1.4 ms. Spectral width was set to 5,995.02 Hz (15 ppm) for standard two-dimensional (2D)^1^H-^1^H correlation spectroscopy (COSY), total correlation spectroscopy (TOCSY), and ^1^H-^13^C heteronuclear single quantum correlation (HSQC). Heteronuclear Multiple Bond Correlation (HMBC) was performed on selected samples (test samples) to allow or confirm the assignment of specific metabolite peaks (**S6 Table**). Signal assignment was achieved by the acquired standard 2D experiments (COSY, TOCSY, HSQC, HMBC) and confirmed by comparison with the literature [87], the Human Metabolome Database [88] and an *in-house* database.

#### ^1^H-NMR data processing and analysis

One-dimensional (1D) NMR spectra were processed and quantified using ACD Lab 1D NMR Manager ver. 12.0 software (Advanced Chemistry Development, Inc., Canada), whereas 2D NMR spectra were processed using Bruker TopSpin ver.3.1 (Bruker BioSpin, Germany). The acquired NMR spectra were manually phased and baseline corrected. Proton spectra were referenced to the chemical shift of the TSP methyl resonance at δ 0.00. Quantification of metabolites was achieved by comparing the integrals of their specific signals with the IS integral and normalized for proton numbers. The concentrations were expressed as μmol/g wet weight of faeces. To avoid possible dilution effects on the levels of faecal metabolites, for each individual metabolite the level was normalized. This procedure allowed for investigation into the relationship between levels of metabolites and OTUs.

### Statistics and correlation analyses

Statistical analysis were carried out using a combination of Sigmaplot 12.0 (Systat Software Inc.) and The UnscrublerX ver. 10.3 (CAMO Software, Oslo, Norway) for univariate and multivariate analyses. To evaluate changes over time, Principal Component Analysis (PCA) was performed on normalized metabolite levels from faecal samples from CS-delivered infants representing the entire set of time points (*i*.*e*., 1–3, 7, 15, 30 days) (**Sheet A in S7 Table**). Data were auto-scaled before analysis [89]. Changes in each individual metabolite over time were evaluated by Kruskal-Wallis test; Dunn’s method was used as a pairwise multiple comparison procedure, and a value of p<0.05 was considered statistically significant. Spearman’s rank correlation test was used to assess the relationships among metabolites (**Sheet A in S8 Table**). Only correlations with a value of p<0.05 were considered significant (**Sheet A in S8 Table**; **Sheets A-E in S9 Table**).

Finally, to assess the relationship among OTUs and metabolites, Spearman's rank correlation test was applied to faecal samples simultaneously analysed by both microarray HITChip and ^1^H-NMR spectroscopy (**Table 1, S1 Table**) by using OTUs and corresponding normalized metabolite levels for each sample. Only correlations with a value of p<0.05 were considered significant (**Sheets A-E in S9 Table**).

## Supporting Information

S1 Fig Graphical representation of variable and stable structures of gut microbiota highlighted in different subsets of babies, stratified for delivery, feeding, perinatal age. Venn diagram represents the OTUs specifically associated with each gut microbiota type or shared by multiple gut microbiota structures.
(TIF)

S2 Fig Carr-Purcell-Meiboom-Gill (CPMG) ^1^H-NMR signal profiles of faecal water obtained from newborn nr. 18 (sample test) at each time point. 1: 2-hydroxy-3-methylbutyrate; 2: butyrate; 3: leucine; 4: isoleucine; 5: propionate; 6: 1,2-propanediol; 7: ethanol; 8: α-and b-fucose; 9: lactate; 10: acetoin; 11: alanine; 12: acetate; 13-N-acetyl moieties 1,2,3; 14: glutamate; 15, pyruvate; 16: succinate; 17: aspartate; 18: creatinine; 19: choline; 20: tyrosine; 21: phenylalanine; 22: formate.
(TIF)

S3 Fig 3D-plot of the scores for the first 3 PCs. The majority of samples at days 1 and 2 are separated from those collected at day 3 along PC2.
(TIF)

S1 Table Succession scheme of stool collection and HITChip microarray-based and 1H-NMR analyses. Baby code, gender, delivery, time course in days, feeding modality, sample ID, HITChip analysis, ^1^H-NMR analysis are reported for each subject.
(DOC)

S2 Table Enrolment metadata. For each mother-baby pair, gender, delivery mode, sample collection time course, feeding modality, gestational age, baby BMI, APGAR score, newborn clinical data, home setting, and socio-economic status are reported.
(XLS)

S3 Table HITChip hybridization profiles and taxonomic cataloguing of newborn gut microbiota. **Sheet A**, Raw microarray HITChip values obtained from all samples; **Sheet B and C**, values of phyla and family relative abundances over the six sampling time points calculated for each newborn.
(XLS)

S4 Table Relative abundance of prevalent phyla and bacterial families in faecal samples obtained from 31 newborns. (DOC)

S5 Table Computation values of OTUs correlations and co-occurrence networks. **Sheet A**, correlation matrix at phylum level; **Sheets A-C**, list of OTUs co-occurrence and grade of interaction for CS 1–3, CS 7–30, and V1-3 days, respectively; **Sheet D**, correlation clusters calculated by MCODE; **Sheet E-F**, list of OTUs found in correlation networks grouped by their uniqueness or commonness in the CS- and V-based groups. Pearson’s test was used to evaluate the correlation among OTUs (statistical significance was assessed with *p*<0.05).
(XLS)

S6 Table ^1^H-NMR metabolite assignment. ^1^H-NMR assignment of 33 metabolites identified and/or quantified in 70 faecal samples from 21 newborns (16 CS- and 5 V-delivered infants) collected at 1–3, 7, 15, and 30 days after delivery.
(DOC)

S7 Table Relative levels of metabolites in 55 faecal samples from 16 CS-delivered infants over 30 days following birth. Data are reported as medians and ranges from first and third quartile and were analysed by Kruskal-Wallis tests. Significant differences in metabolite levels (p<0.05) are reported between day 1 and 2 (**a**), 1 and 3 (**b**), 1 and 7 (**c**), 1 and 15 (**d**), 1 and 30 (**e**), 2 and 3 (**f**), 2 and 7 (**g**), 2 and 15 (**h**), 2 and 30 (**i**), 3 and 7 (**j**), 3 and 15 (**k**), 3 and 30 (**l**), 7 and 15 (**m**), 7 and 30 (**n**), and 15 and 30 (**o**).
(DOC)

S8 Table Metabolite quantity matrix for CS-and V-delivered babies. Metabolites quantified in the samples listed in the A column are reported as normalized quantities (concentration of μmol/g of wet weight of faeces).
(XLS)

S9 Table Spearman's rank correlation for metabolite-metabolite and phylum-metabolite. Data are reported as Spearman's rank correlation coefficient and p-value for each metabolite-metabolite and metabolite–OTU correlation.
(XLS)
