# Supplementary figures and images for: Phylogenetic and Metabolic Tracking of Gut Microbiota during Perinatal Development
Source: PLoS One. 2015 Sep 2;10(9):e0137347. doi: 10.1371/journal.pone.0137347 (PMC4557834; doi:10.1371/journal.pone.0137347)

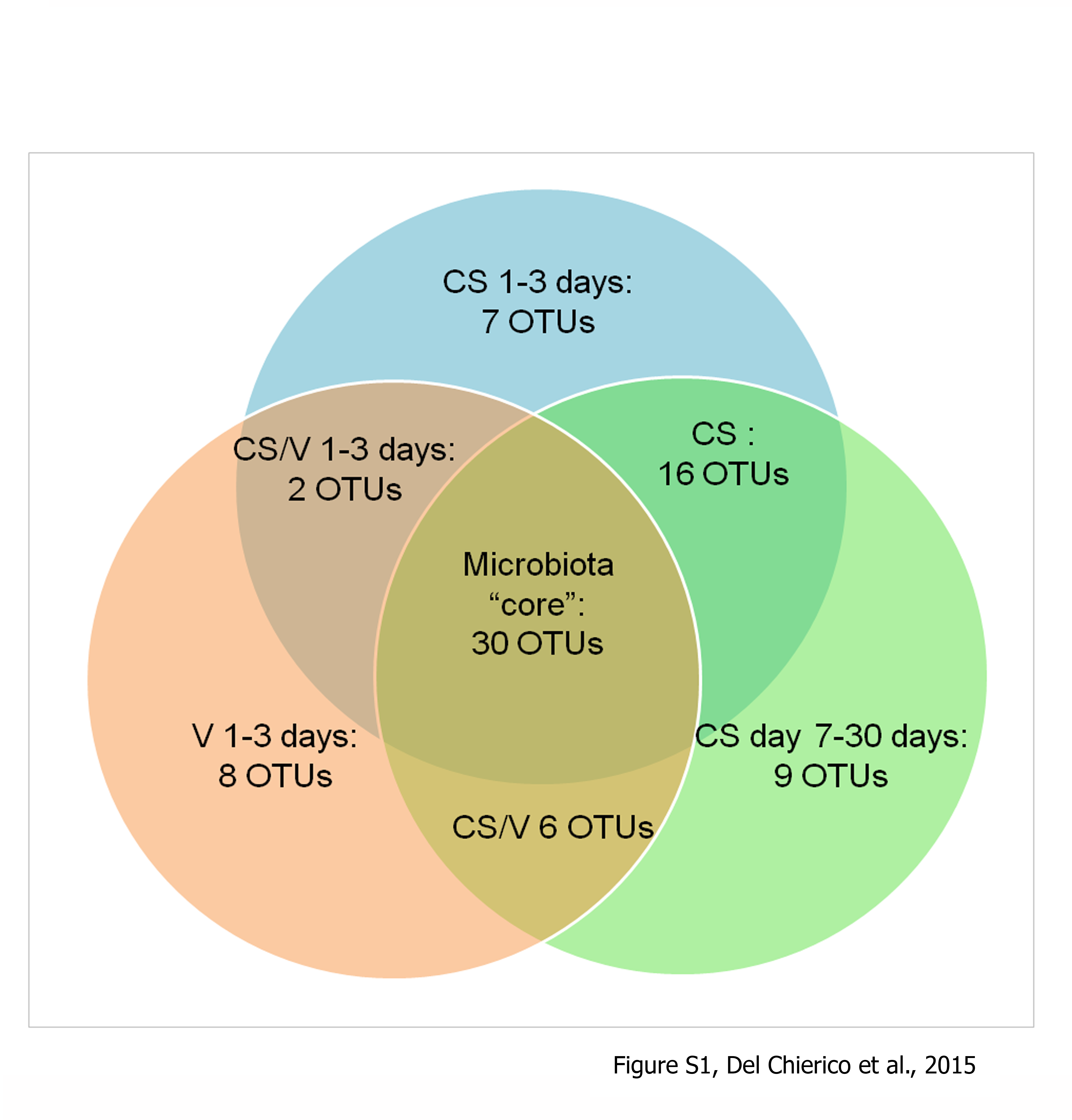

Supplement: S1 Fig — Venn diagram represents the OTUs specifically associated with each gut microbiota type or shared by multiple gut microbiota structures. (TIF) [file pone.0137347.s001.tif]

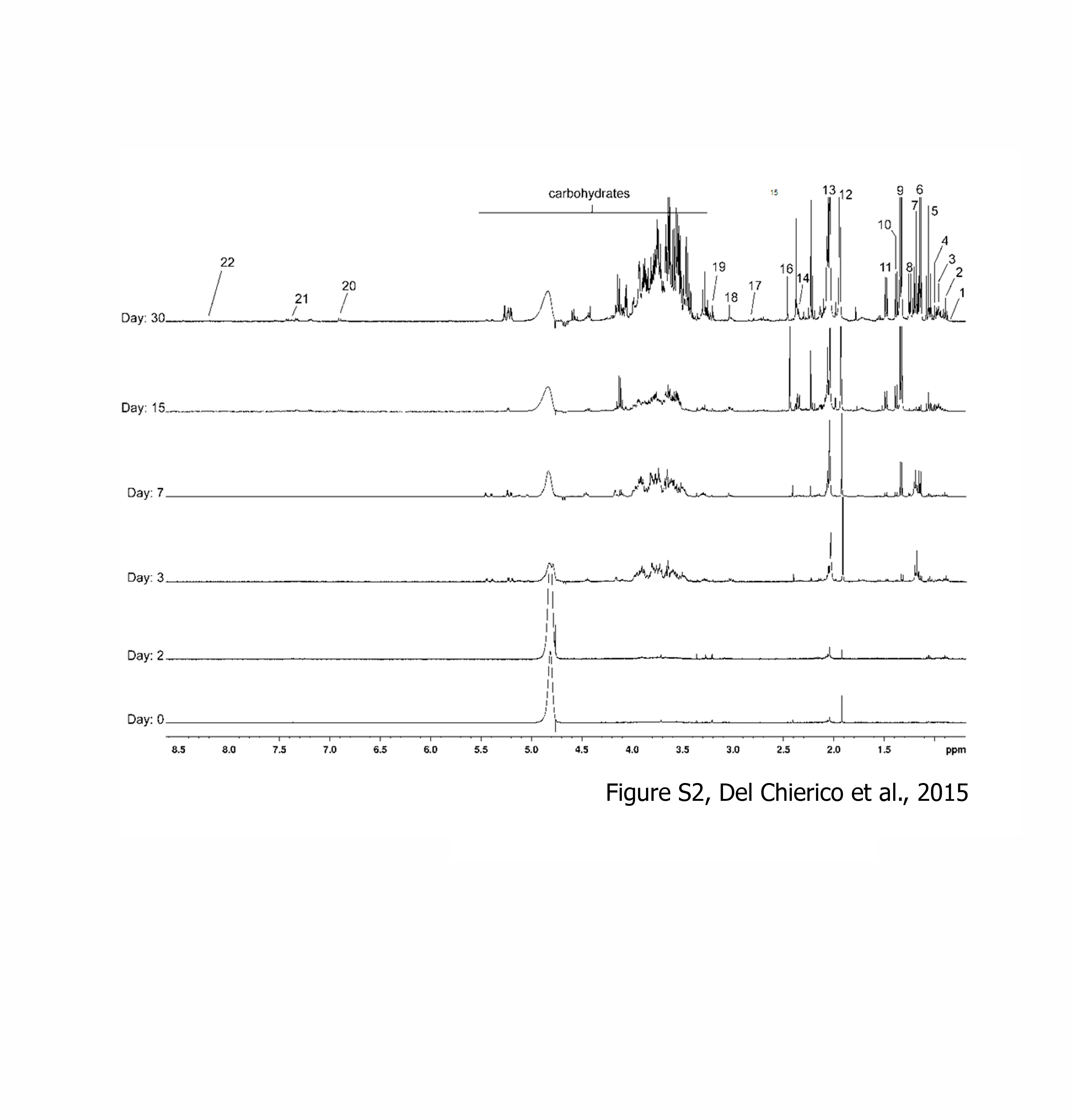

Supplement: S2 Fig — 1: 2-hydroxy-3-methylbutyrate; 2: butyrate; 3: leucine; 4: isoleucine; 5: propionate; 6: 1,2-propanediol; 7: ethanol; 8: α-and b-fucose; 9: lactate; 10: acetoin; 11: alanine; 12: acetate; 13-N-acetyl moieties 1,2,3; 14: glutamate; 15, pyruvate; 16: succinate; 17: aspartate; 18: creatinine; 19: choline; 20: tyrosine; 21: phenylalanine; 22: formate. (TIF) [file pone.0137347.s002.tif]

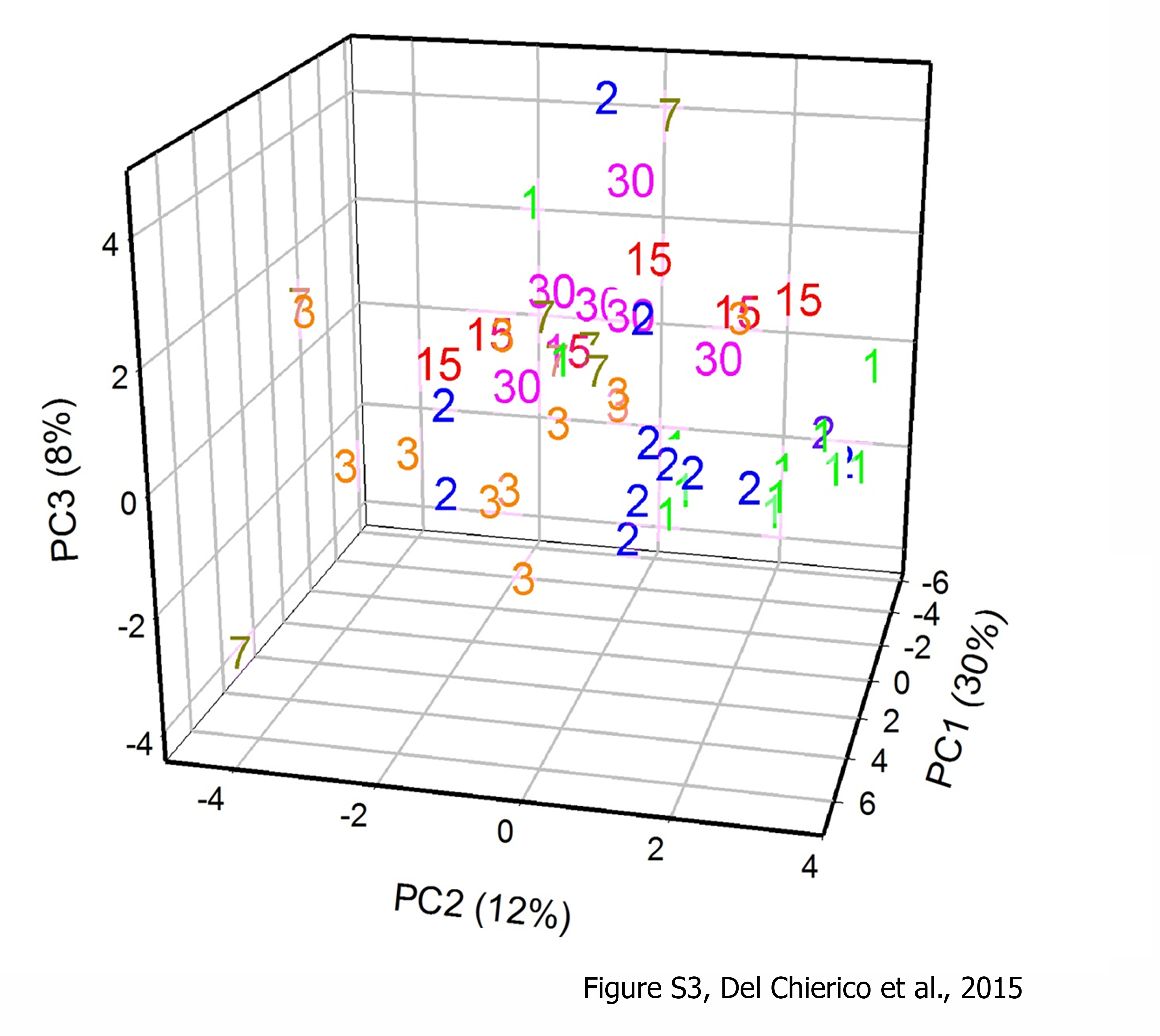

Supplement: S3 Fig — The majority of samples at days 1 and 2 are separated from those collected at day 3 along PC2. (TIF) [file pone.0137347.s003.tif]
