# Supplementary material for: Phylogenetic and Metabolic Tracking of Gut Microbiota during Perinatal Development
Source: PLoS One. 2015 Sep 2;10(9):e0137347. doi: 10.1371/journal.pone.0137347 (PMC4557834; doi:10.1371/journal.pone.0137347)
Supplement: S1 Table — Baby code, gender, delivery, time course in days, feeding modality, sample ID, HITChip analysis, 1H-NMR analysis are reported for each subject. (DOC) [file pone.0137347.s004.doc]

**S1 Table.** Succession scheme of stool collection and HITChip microarray-based and 1H-NMR analyses. Baby code, gender, delivery, time course in days, feeding modality, sample ID, HITChip analysis, 1H-NMR analysis are reported for each subject.

| **Baby code** | **Gender** | **Delivery^1^** | **Time course (days)** | **Feeding^2^** | **Sample ID** | **HITChip^3^** | **^1^H-NMR** |
| --- | --- | --- | --- | --- | --- | --- | --- |
| 1 | M | CS | 1 | FM | 1_1_FM |  | **×** |
|  |  |  | 2 | FM | 1_2_FM |  | **×** |
|  |  |  | 3 | FM | 1_3_FM | **×** | **×** |
|  |  |  | 7 | BM | 1_7_BM | **×** | **×** |
| 2 | M | V | 1 | FM | 2_1_FM |  | **×** |
|  |  |  | 2 | FM | 2_2_FM |  | **×** |
|  |  |  | 3 | FM | 2_3_FM | **×** | **×** |
| 3 | M | CS | 1 | FM | 3_1_FM |  |  |
|  |  |  | 2 | FM | 3_2_FM | **×** |  |
|  |  |  | 3 | FM | 3_3_FM |  |  |
|  |  |  | 7 | BM | 3_7_BM | **×** |  |
|  |  |  | 15 | BM | 3_15_BM | **×** |  |
|  |  |  | 30 | BM | 3_30_BM | **×** |  |
| 4 | F | CS | 1 | FM | 4_1_FM |  | **×** |
|  |  |  | 2 | FM | 4_2_FM |  | **×** |
| 5 | M | CS | 1 | FM | 5_1_FM | **×** | **×** |
|  |  |  | 2 | FM | 5_2_FM |  | **×** |
|  |  |  | 3 | FM | 5_3_FM | **×** | **×** |
| 6 | F | CS | 1 | FM | 6_1_FM |  |  |
|  |  |  | 2 | FM | 6_2_FM |  |  |
|  |  |  | 3 | FM | 6_3_FM | **×** | **×** |
|  |  |  | 7 | BM | 6_7_BM |  | **×** |
|  |  |  | 15 | BM | 6_15_BM | **×** | **×** |
|  |  |  | 30 | BM | 6_30_BM | **×** | **×** |
| 7 | F | CS | 1 | FM | 7_1_FM |  | **×** |
|  |  |  | 2 | FM | 7_2_FM | **×** | **×** |
|  |  |  | 3 | FM | 7_3_FM | **×** | **×** |
|  |  |  | 7 | fM | 7_7_fM |  | **×** |
|  |  |  | 15 | fM | 7_15_fM | **×** | **×** |
|  |  |  | 30 | fM | 7_30_fM | **×** | **×** |
| 8 | M | CS | 1 | FM | 8_1_FM |  |  |
|  |  |  | 2 | FM | 8_2_FM | **×** |  |
|  |  |  | 3 | FM | 8_3_FM | **×** |  |
|  |  |  | 7 | BM | 8_7_BM | **×** | **×** |
|  |  |  | 15 | MM | 8_15_MM | **×** | **×** |
| 9 | M | CS | 1 | FM | 9_1_FM |  |  |
|  |  |  | 2 | FM | 9_2_FM |  |  |
|  |  |  | 3 | FM | 9_3_FM | **×** |  |
|  |  |  | 7 | BM | 9_7_BM | **×** |  |
| 10 | M | CS | 1 | FM | 10_1_FM |  |  |
|  |  |  | 2 | FM | 10_2_FM |  |  |
|  |  |  | 3 | FM | 10_3_FM | **×** |  |
|  |  |  | 7 | BM | 10_7_BM |  | **×** |
|  |  |  | 15 | BM | 10_15_BM |  |  |
|  |  |  | 30 | BM | 10_30_BM | **×** | **×** |
| 11 | M | CS | 1 | FM | 11_1_FM | **×** | **×** |
|  |  |  | 2 | FM | 11_2_FM | **×** | **×** |
|  |  |  | 3 | FM | 11_3_FM |  |  |
|  |  |  | 7 | MM | 11_7_MM | **×** |  |
| 12 | F | CS | 1 | FM | 12_1_FM |  |  |
|  |  |  | 2 | FM | 12_2_FM |  |  |
|  |  |  | 3 | FM | 12_3_FM | **×** | **×** |
| 13 | M | CS | 1 | FM | 13_1_FM |  |  |
|  |  |  | 2 | FM | 13_2_FM |  |  |
|  |  |  | 3 | FM | 13_3_FM | **×** |  |
| 14 | M | CS | 1 | FM | 14_1_FM |  | **×** |
|  |  |  | 2 | FM | 14_2_FM |  | **×** |
|  |  |  | 3 | FM | 14_3_FM |  | **×** |
|  |  |  | 7 | BM | 14_7_BM | **×** | **×** |
|  |  |  | 15 | BM | 14_15_BM | **×** | **×** |
|  |  |  | 30 | BM | 14_30_BM | **×** | **×** |
| 15 | M | CS | 1 | FM | 15_1_M |  |  |
| 16 | M | CS | 1 | FM | 16_1_FM | **×** | **×** |
|  |  |  | 2 | FM | 16_2_FM |  | **×** |
|  |  |  | 3 | FM | 16_3_FM |  | **×** |
|  |  |  | 7 | BM | 16_7_BM | **×** | **×** |
| 17 | M | CS | 1 | FM | 17_1_FM | **×** |  |
|  |  |  | 2 | FM | 17_2_FM |  |  |
|  |  |  | 3 | FM | 17_3_FM | **×** |  |
|  |  |  | 7 | BM | 17_7_BM | **×** |  |
|  |  |  | 15 | BM | 17_15_BM | **×** |  |
|  |  |  | 30 | fM | 17_30_fM | **×** |  |
| 18 | F | CS | 1 | FM | 18_1_FM | **×** | **×** |
|  |  |  | 2 | FM | 18_2_FM | **×** | **×** |
|  |  |  | 3 | FM | 18_3_FM | **×** | **×** |
|  |  |  | 7 | BM | 18_7_BM | **×** | **×** |
|  |  |  | 15 | BM | 18_15_BM | **×** | **×** |
|  |  |  | 30 | MM | 18_30_MM | **×** | **×** |
| 19 | F | CS | 1 | FM | 19_1_FM |  |  |
|  |  |  | 2 | FM | 19_2_FM |  |  |
|  |  |  | 3 | FM | 19_3_FM | **×** |  |
|  |  |  | 7 | BM | 19_7_BM | **×** |  |
|  |  |  | 15 | BM | 19_15_BM | **×** |  |
|  |  |  | 30 | MM | 19_30_MM | **×** |  |
| 20 | M | CS | 1 | FM | 20_1_FM | **×** |  |
|  |  |  | 2 | FM | 20_2_FM |  |  |
|  |  |  | 3 | FM | 20_3_FM | **×** |  |
| 21 | M | CS | 1 | FM | 21_1_FM |  | **×** |
|  |  |  | 2 | FM | 21_2_FM | **×** | **×** |
|  |  |  | 3 | FM | 21_3_FM | **×** | **×** |
| 22 | F | CS | 1 | FM | 22_1_FM |  | **×** |
|  |  |  | 2 | FM | 22_2_FM |  | **×** |
|  |  |  | 3 | FM | 22_3_FM | **×** | **×** |
|  |  |  | 7 | BM | 22_7_BM |  |  |
|  |  |  | 15 | BM | 22_15_BM | **×** | **×** |
|  |  |  | 30 | BM | 22_30_BM | **×** | **×** |
| 23 | M | CS | 1 | FM | 23_1_FM |  |  |
|  |  |  | 2 | FM | 23_2_FM |  |  |
|  |  |  | 3 | FM | 23_3_FM | × |  |
| 24 | M | CS | 1 | FM | 24_1_FM |  |  |
|  |  |  | 2 | FM | 24_2_FM |  |  |
|  |  |  | 3 | FM | 24_3_FM |  |  |
| 25 | F | V | 1 | FM | 25_1_FM |  | × |
|  |  |  | 2 | FM | 25_2_FM | **×** | **×** |
|  |  |  | 3 | FM | 25_3_FM | **×** | **×** |
| 26 | M | V | 1 | FM | 26_1_FM |  |  |
|  |  |  | 2 | FM | 26_2_FM |  |  |
|  |  |  | 3 | FM | 26_3_FM | **×** |  |
| 27 | F | V | 1 | FM | 27_1_FM |  | **×** |
|  |  |  | 2 | FM | 27_2_FM |  | **×** |
|  |  |  | 3 | FM | 27_3_FM | **×** | **×** |
| 28 | F | V | 1 | FM | 28_1_FM | **×** | **×** |
|  |  |  | 2 | FM | 28_2_FM | **×** | **×** |
|  |  |  | 3 | FM | 28_3_FM | **×** |  |
| 29 | F | CS | 1 | FM | 29_1_FM | **×** | **×** |
|  |  |  | 2 | FM | 29_2_FM |  | **×** |
|  |  |  | 3 | FM | 29_3_FM | **×** | **×** |
| 30 | M | CS | 1 | FM | 30_1_M |  | **×** |
|  |  |  | 2 | FM | 30_2_FM |  | **×** |
| 31 | M | V | 1 | FM | 31_1_FM | **×** | × |
|  |  |  | 2 | FM | 31_2_FM |  | **×** |
|  |  |  | 3 | FM | 31_3_FM | **×** | **×** |
|  |  |  | 7 | BM | 31_7_BM | **×** | **×** |

^1^CS. caesarean section delivery; V. vaginal delivery; ^2^FM. first-milk or colostrum; BM. breast-milk; fM. formula-milk; MM. mixed-milk, BM plus fM; ^3^na. not available; ^4^BMI. body mass index; ^5^APGAR, appearance, pulse, grimace; activity, respiration
