# Supplementary material for: Phylogenetic and Metabolic Tracking of Gut Microbiota during Perinatal Development
Source: PLoS One. 2015 Sep 2;10(9):e0137347. doi: 10.1371/journal.pone.0137347 (PMC4557834; doi:10.1371/journal.pone.0137347)
Supplement: S4 Table — (DOC) [file pone.0137347.s007.doc]

**Table S4**. Relative abundance of prevalent phyla and bacterial families in faecal samples obtained from 31 newborns.

| **Taxon** | **Time course** | | |  | **Time course** | | |  | **Time course** |
| --- | --- | --- | --- | --- | --- | --- | --- | --- | --- |
|  | **CS-1 days** | **CS-2 days** | **CS-3 days** |  | **CS-7 days** | **CS-15 days** | **CS-30 days** |  | **V-3 days** |
|  | **Median (IQR^a^)** | **Median (IQR)** | **Median (IQR)** |  | **Median (IQR)** | **Median (IQR)** | **Median (IQR)** |  | **Median (IQR)** |
| **Actinobacteria** | **3.4 (0.97-23.23)** | **1.12 (0.4-5.0)** | **0.56 (0.46-1.86)** |  | **0.92 (0.41-12.48)** | **5.74 (0.43-24.48)** | **7.80 (1.0-46.7)** |  | **0.56 (0.35-1.99)** |
| Bifidobacteriaceae | 0.66 (0.36-1.6) | 0.31 (0.20-0.50) | 0.32 (0.21-0.40) |  | 0.42 (0.21-8.95) | 5.59 (0.22-24.3) | 7.71 (0.82-46.58) |  | 0.40 (0.14-1.50) |
| Propionibacteriaceae | 2.40 (0.09-22.49) | 0.40 (0.01-3.97) | 0.08 (0.01-0.69) |  | 0.02 (0.01-0.10) | 0.02 (0.01-0.04) | 0.02(0.02-0.03) |  | 0.05 (0.03-0.21) |
|  |  |  |  |  |  |  |  |  |  |
| **Bacteroidetes** | **0.89 (0.84-0.99)** | **1.13 (0.77-1.76)** | **0.64 (0.50-1.27)** |  | **0.63 (0.49-0.78)** | **0.67 (0.56-0.81)** | **0.70 (0.61-0.93)** |  | **6.90 (1.13-21.2)** |
| Bacteroidaceae | 0.42 (0.41-0.49) | 0.52 (0.36-0.82) | 0.30 (0.23-0.61) |  | 0.31 (0.24-0.37) | 0.32 (0.27-0.44) | 0.34 (0.30-0.49) |  | 6.47 (0.64-20.35) |
| Prevotellaceae | 0.25 (0.20-0.42) | 0.32 (0.24-0.51) | 0.19 (0.15-0.30) |  | 0.19 (0.14-0.24) | 0.20 (0.16-0.22) | 0.19 (0.18-0.23) |  | 0.38 (0.20-0.56) |
|  |  |  |  |  |  |  |  |  |  |
| **Firmicutes** | **26.4 (17.4-31.60)** | **62.3 (24.3-94.5)** | **69.73 (19.58-92.46)** |  | **53.91 (29.97-88.57)** | **38.93 (25.28-68.60)** | **35.8 (24.3-51.97)** |  | **26.40 (7.16-58.30)** |
| Enterococcaceae | 0.38 (0.07-1.95) | 2.15 (0.70-3.21) | 2.47 (0.49-3.77) |  | 0.58 (0.30-2.24) | 0.47 (0.26-5.41) | 0.27 (0.15-14.35) |  | 1.74 (0.10-2.93) |
| Lactobacillaceae | 0.32 (0.18-0.42) | 0.29 (0.21-2.74) | 0.25 (0.21-0.34) |  | 0.21 (0.17-0.32) | 0.54 (0.17-2.36) | 0.35 (0.17-6.37) |  | 0.19 (0.15-0.24) |
| Staphylococcaceae | 0.07 (0.05-23.3) | 18.6 (0.38-35.44) | 4.34 (0.64-14.73) |  | 1.68 (0.19-11.61) | 0.41 (0.20-1.20) | 0.17 (0.13-0.38) |  | 0.43 (0.02-0.96) |
| Streptococcaceae | 4.28 (1.21-11.4) | 7.96 (3.35-38.34) | 4.24 (0.58-11.46) |  | 11.77 (5.23-39.04) | 15.4 (9.58-27.59) | 10.91 (8.85-12.30) |  | 0.60 (0.22-8.23) |
| Clostridiaceae | 3.04 (1.58-17.1) | 4.37 (1.97-10.55) | 3.98 (1.47-7.20) |  | 12.51 (4.78-21.10) | 10.28 (4.53-13.79) | 7.50 (5.62-9.39) |  | 1.84 (0.99-18.68) |
| Ruminococcaceae | 0.73 (0.62-0.84) | 1.17 (0.84-1.40) | 0.89 (0.64-1.56) |  | 0.80 (0.64-1.14) | 0.92 (0.73-1.17) | 0.82 (0.67-1.06) |  | 0.75 (0.47-1.08) |
| Peptostreptococcaceae | 0.22 (0.12-0.27) | 0.41 (0.25-1.25) | 1.52 (0.22-27.24) |  | 1.19 (0.21-6.44) | 1.02 (0.17-1.75) | 0.54 (0.32-2.92) |  | 1.67 (0.08-29.08) |
| Lachnospiraceae | 1.73 (1.18-6.37) | 3.60 (1.24-4.60) | 2.54 (1.21-8.29) |  | 3.24 (1.45-4.06) | 1.85 (1.41-3.23) | 1.27 (1.22-2.25) |  | 1.45 (0.75-4.86) |
|  |  |  |  |  |  |  |  |  |  |
| **Proteobacteria** | **63.13 (36.5-70.8)** | **34.1 (2.33-61.60)** | **12.94 (2.26-71.31)** |  | **30.40 (2.07-61.60)** | **34.2 (10.70-65.23)** | **40.8 (11.18-61.21)** |  | **63.4 (14.96-83.88)** |
| Enterobacteriaceae | 36.2 (10.13-62.9) | 8.77 (1.71-55.42) | 5.09 (1.41-70.59) |  | 29.82 (1.50-61.08) | 33.55 (9.35-64.51) | 40.44 (10.83-60.67) |  | 62.73 (2.98-83.40) |
| Oxalobacteraceae | 0.46 (0.12-3.48) | 0.16 (0.03-3.65) | 0.13 (0.05-0.30) |  | 0.10 (0.08-0.17) | 0.01 (0.05-0.12) | 0.06 (0.05-0.08) |  | 0.06 (0.03-0.12) |
| Pseudomonadaceae | 0.02 (0.01-0.06) | 0.01 (0.01-0.02) | 0.01 (0.01-0.02) |  | 0.01 (0.01-0.02) | 0.01 (0.01-0.02) | 0.01 (0.01-0.02) |  | 0.03 (0.01-3.80) |
| Sutterellaceae | 0.14 (0.07-0.29) | 0.08 (0.06-0.74) | 0.07 (0.06-0.09) |  | 0.07 (0.05-0.09) | 0.06 (0.05-0.09) | 0.07 (0.05-0.09) |  | 0.06 (0.05-0.08) |
| Xanthomonadaceae | 0.14 (0.06-0.21) | 0.08 (0.04-0.26) | 0.14 (0.04-0.37) |  | 0.02 (0.01-0.03) | 0.02 (0.01-0.04) | 0.02 (0.01-0.10) |  | 0.04 (0.01-0.50) |
|  |  |  |  |  |  |  |  |  |  |
| **Verrucomicrobia** | **0.28 (0.09-3.70)** | **1.5 (0.05-1.84)** | **0.13 (0.07-0.80)** |  | **1.06 (0.52-2.08)** | **0.42 (0.18-1.61)** | **0.36 (0.22-0.85)** |  | **0.07 (0.06-1.81)** |
| Verrucomicrobiae | 0.28 (0.09-3.70) | 1.5 (0.05-1.84) | 0.13 (0.07-0.80) |  | 1.06 (0.52-2.08) | 0.42 (0.18-1.61) | 0.36 (0.22-0.85) |  | 0.07 (0.06-1.81) |
|  |  |  |  |  |  |  |  |  |  |
| **Other phyla** | **nd** | **nd** | **nd** |  | **nd** | **nd** | **nd** |  | **nd** |
| Other bacterial families | 10.05 (2.50-22.27) | 1.99 (8.32-17.12) | 3.08 (2.21-6.41) |  | 2.34 (1.32-7.64) | 1.84 (1.43-10.37) | 2.24 (1.38-6.32) |  | 3.02 (1.24-4.51) |

^a^ IQR, interquantile range; nd, not determined.
