# Supplementary material for: Phylogenetic and Metabolic Tracking of Gut Microbiota during Perinatal Development
Source: PLoS One. 2015 Sep 2;10(9):e0137347. doi: 10.1371/journal.pone.0137347 (PMC4557834; doi:10.1371/journal.pone.0137347)
Supplement: S6 Table — 1H-NMR assignment of 33 metabolites identified and/or quantified in 70 faecal samples from 21 newborns (16 CS- and 5 V-delivered infants) collected at 1–3, 7, 15, and 30 days after delivery. (DOC) [file pone.0137347.s009.doc]

**Table S6.** ^1^H-NMR assignment of 34 metabolites quantified in 70 fecal samples from 21 newborns (16 CS- and 5 V-delivered infants) collected at 1-3, 7, 15, 30 days after delivery

| **N°** | **Metabolite** | **Chemical Shift δ (ppm)** |
| --- | --- | --- |
| 1 | U1 | 0.79 (d) |
| 2 | U2 | 0.83 |
| 3 | 2-hydroxy-3-methylbutyrate | 0.85 (d), 0.96 (d) |
| 4 | Isocaproic Acid | 0.89 (d), 1.45 (m), 2.18 (m) |
| 5 | Isovalerate | 0.91 (d), 2.06 |
| 6 | Isoleucine | 0.94 (d), 1.02 (t), 1.27, 1.47, 1.99, 3.68 |
| 7 | Leucine | 0.96, 1.72, 3.73 |
| 8 | 1,2-propanediol | 1.14 (d), 3.43 (dd), 3.54 (dd), 3.87 |
| 9 | Ethanol | 1.19(t), 3.64(q) |
| 10 | Fucose | α-Fuc: 1.20 (d), 4.21 (d); β-Fuc 1.25 (d), 3.82(q) |
| 11 | 3-Hydroxyisovalerate | 1.25 (s) |
| 12 | Lactate | 1.33 (d), 4.11 |
| 13 | Acetoin | 1.38 (d), 2.22, 4.43 (q) |
| 14 | Alanine | 1.48 (d), 3.79 |
| 15 | Butyrate | 0.90 (t), 1.57 (m), 2.16 |
| 16 | Acetate | 1.90 (s) |
| 17 | N-acetyl moiety 1 | 2.04 (s) |
| 18 | N-acetyl moiety 2 | 2.05 (s) |
| 19 | N-acetyl moiety 3 | 2.06 (s) |
| 20 | Glutamate | 2.04, 2.12, 2.36 (dd), 3.75 |
| 21 | Propionate | 1.05 (t), 2,19 (q) |
| 22 | Succinate | 2.40 (s) |
| 23 | Dimethylamine | 2.73 (s) |
| 24 | Aspartate | 2.68 (dd), 2.82 (dd), 3.90 (m) |
| 25 | Trimethylamine | 2.90 (s) |
| 26 | Creatinine | 3.05 (s), 4.07 (s) |
| 27 | U3 | 3.08, 3.57 (dd) |
| 28 | Malonate | 3.14 (s) |
| 29 | Choline | 3.21 (s) |
| 30 | Methanol | 3.36 (s) |
| 31 | Tyrosine | 6.90 (pd), 7.20 (pd) |
| 32 | Phenylalanine | 7.30-7.40 (ov) |
| 33 | Formate | 8.47 (s) |
| Symbols: (s): singlet; (d): doublet; (dd) doublet of doublets; (t) triplet; (q) quartet; (m) multiplet; (pd) pseudo-doublet; (ov) overlapped signals. | | |
