# Supplementary material for: Phylogenetic and Metabolic Tracking of Gut Microbiota during Perinatal Development
Source: PLoS One. 2015 Sep 2;10(9):e0137347. doi: 10.1371/journal.pone.0137347 (PMC4557834; doi:10.1371/journal.pone.0137347)
Supplement: S7 Table — Data are reported as medians and ranges from first and third quartile and were analysed by Kruskal-Wallis tests. Significant differences in metabolite levels (p<0.05) are reported between day 1 and 2 (a), 1 and 3 (b), 1 and 7 (c), 1 and 15 (d), 1 and 30 (e), 2 and 3 (f), 2 and 7 (g), 2 and 15 (h), 2 and 30 (i), 3 and 7 (j), 3 and 15 (k), 3 and 30 (l), 7 and 15 (m), 7 and 30 (n), and 15 and 30 (o). (DOC) [file pone.0137347.s010.doc]

| ***Metabolite*** | ***Day 1*** | ***Day 2*** | ***Day 3*** | ***Day 7*** | ***Day 15*** | ***Day 30*** |
| --- | --- | --- | --- | --- | --- | --- |
| U1 | 0.821 (0.541-0.859) | 0.760 (0.594-1.486) | 1.017 (0.766-1.823) | 0.280 (0.166-0.577) ^g,j^ | 0.064 (-0.077-0.155)^d,h,k^ | 0.186 (0.040-0.383) ^i,l^ |
| U2 | 0.301 (0.243-0.405) | 0.563 (0.303-0.615) | 0.632 (0.364-0.707) | 0.132 (0.116-0.235) ^j^ | 0.081 (0.071-0.223)^k^ | 0.097 (0.062-0.165) ^l^ |
| 2-hydroxy-3-methylbutyrate | 0.487 (0.363-0.674) | 0.609 (0.445-0.913) | 0.747 (0.675-0.943) | 0.408 (0.296-0.497) | 0.322 (0.167-0.463)^k^ | 0.166 (0.075-0.323) ^i,l^ |
| Isocaproate | 0.362 (0.281-0.393) | 0.474 (0.372-0.529) | 0.619 (0.499-0.872) | 0.200 (0.174-0.446) ^j^ | 0.281 (0.186-0.451) | 0.158 (0.053-0.237) ^l^ |
| Isovalerate | 1.083 (0.918-1.267) | 1.231 (0.883-1.284) | 1.414 (1.132-1.874) | 0.824 (0.608-1.005) | 0.528 (0.516-0.597)^k^ | 0.369 (0.310-0.533) ^i,l^ |
| Isoleucine | 1.173 (0.998-1.520) | 1.284 (1.166-1.561) | 1.399 (1.092-1.659) | 0.553 (0.535-0.774) ^g^ | 0.553 (0.316-0.753)^h,k^ | 0.415 (0.328-0.547) ^i,l^ |
| Leucine | 1.274 (1.110-1.514) | 1.786 (1.343-2.153) | 1.453 (1.233-1.955) | 0.888 (0.728-1.295) | 0.660 (0.548-0.973)^h^ | 0.599 (0.473-0.771) ^i,l^ |
| 1,2-propanediol | 1.177 (0.799-1.278) | 1.342 (1.056-1.823) | 1.452 (0.853-1.980) | 1.315 (0.727-2.583) | 1.596 (1.396-2.225) | 3.446 (1.644-7.879) |
| Ethanol | 2.237 (2.138-2.426) | 2.485 (2.100-2.963) | 2.122 (1.849-2.828) | 1.661 (0.732-3.252) | 1.814 (1.150-2.387) | 3.446 (2.382-3.677) |
| Fucose | 0.854 (0.810-1.083) | 0.956 (0.775-1.126) | 0.816 (0.708-1.254) | 0.654 (0.604-0.739) | 0.794 (0.350-0.955) | 0.671 (0.493-0.898) |
| 3-hydroxyisovalerate | 0.425 (0.267-0.531) | 0.282 (0.229-1.890) | 0.368 (0.132-0.754) | 0.272 (0.159-0.470) | 0.063 (-0.442-0.070) | 0.041 (-0.025-0.182) |
| Lactate | 1.821 (1.557-2.213) | 2.355 (1.691-2.934) | 2.177 (1.934-2.410) | 2.379 (1.511-7.030) | 1.035 (0.897-1.787) | 2.532 (1.933-5.315) |
| Acetoin | 0.909 (0.731-1.020) | 0.959 (0.856-1.176) | 0.984 (0.851-1.177) | 1.267 (0.578-2.331) | 0.900 (0.746-1.260) | 1.804 (0.628-1.845) |
| Alanine | 0.910 (0.822-1.097) | 1.260 (1.048-1.473) | 1.380 (1.278-1.524) | 1.428 (1.174-1.769) | 0.794 (0.765-1.937) | 1.991 (1.522-2.031) |
| Butyrate | 1.435 (1.247-1.651) | 1.581 (1.385-2.199) | 1.862 (1.540-3.676) | 1.550 (0.766-1.796) | 0.739 (0.727-1.961) | 0.598 (0.498-1.016) |
| Acetate | 1.319 (1.066-1.615) | 2.139 (1.307-4.991) | 20.122 (9.950-32.464) | 28.918 (21.802-37.703)^c^ | 30.595 (29.326-32.908)^d^ | 42.060 (41.521-50.219)^e,i^ |
| N-acetyl moiety 1 | 24.377 (19.776-25.561) | 21.459 (12.319-23.438) | 13.317 (8.993-16.070)^b^ | 11.844 (6.078-16.814)^c^ | 12.217 (9.967-15.265)^d^ | 7.196 (3.597-9.603)^e,i^ |
| N-acetyl moiety 2 | 11.405 (9.242-22.642) | 10.142 (5.483-15.148) | 2.781 (0.456-7.204)^b^ | 1.692 (1.117-3.134)^c,g^ | 4.154 (3.854-6.018) | 2.739 (1.952-3.338)^e^ |
| N-acetyl moiety 3 | 2.547 (1.651-6.365) | 2.515 (1.564-4.124) | 3.481 (2.451-4.725) | 2.915 (1.105-6.336) | 5.645 (5.522-5.648) | 3.071 (2.339-4.801) |
| Glutamate | 2.689 (2.495-3.025) | 2.822 (2.426-3.090) | 2.205 (1.796-2.806) | 0.984 (0.875-1.106)^c,g^ | 0.952 (0.703-1.132)^d,h^ | 0.549 (0.519-0.763)^e,i^ |
| Propionate | 9.724 (8.774-11.863) | 10.011 (9.705-11.130) | 8.537 (6.993-10.597) | 3.252 (2.808-3.988)^c,g,j^ | 5.032 (3.972-5.195)^h^ | 4.929 (3.099-5.376) |
| Succinate | 0.380 (0.364-0.496) | 0.559 (0.532-0.593) | 0.674 (0.568-1.167) | 0.384 (0.312-0.535) | 0.200 (0.176-0.559) | 1.151 (0.186-3.766) |
| Dimethylamine | 0.168 (0.136-0.195) | 0.164 (0.149-0.195) | 0.126 (0.121-0.172) | 0.059 (0.025-0.061)^c,g,j^ | 0.053 (0.048-0.073)^d,h,k^ | 0.036 (0.025-0.053)^e,i,l^ |
| Aspartate | 2.638 (2.054-3.020) | 2.454 (1.149-2.735) | 1.790 (1.610-2.179) | 1.276 (0.882-1.416)^c^ | 0.331 (0.157-0.935)^d,h,k^ | 0.360 (0.240-0.456)^e,i,l^ |
| Trimethylamine | 0.057 (0.038-0.065) | 0.059 (0.035-0.069) | 0.059 (0.056-0.070) | 0.029 (0.009-0.045) | 0.011 (-0.002-0.016) | 0.011 (0.007-0.065) |
| Creatinine | 0.594 (0.409-0.760) | 0.460 (0.428-0.603) | 0.825 (0.583-0.953) | 0.449 (0.317-0.563) | 0.441 (0.263-0.540) | 0.300 (0.235-0.422)^l^ |
| U3 | 3.409 (2.597-4.215) | 3.021 (2.315-4.421) | 2.416 (1.601-3.252) | 0.647 (0.412-0.899)^c,g^ | 0.518 (0.284-0.639)^d,h^ | 0.407 (0.179-0.449)^e,i,l^ |
| Malonate | 1.513 (1.159-1.790) | 1.465 (1.224-1.847) | 1.292 (0.890-1.461) | 0.469 (0.235-0.708)^c,g,j^ | 0.395 (0.369-0.788)^d,h^ | 0.270 (0.266-0.620)^e,i^ |
| Choline | 0.516 (0.344-0.708) | 0.829 (0.479-1.381) | 0.579 (0.436-0.981) | 0.143 (0.107-0.173)^g,j^ | 0.186 (0.108-0.375) | 0.131 (0.122-0.135)^i,l^ |
| Methanol | 1.594 (0.927-1.795) | 1.390 (1.140-2.797) | 1.409 (0.804-1.906) | 0.928 (0.731-2.192) | 1.291 (1.025-1.801) | 0.516 (0.454-0.520) |
| Tyrosine | 0.044 (0.033-0.384) | 0.130 (-0.079-0.789) | 0.753 (0.525-1.010) | 0.861 (0.481-1.585) | 0.670 (0.339-0.994) | 0.480 (0.204-0.490) |
| Phenylalanine | 5.267 (3.557-7.528) | 5.862 (2.965-8.970) | 7.497 (4.768-7.608) | 5.372 (2.617-5.621) | 2.351 (1.734-3.410) | 2.008 (1.305-2.590) |
| Formate | 0.088 (-0.015-0.231) | 0.049 (-0.139-0.807) | 0.131 (-0.040-0.445) | 0.454 (0.166-0.603) | 0.130 (0.028-0.236) | 0.099 (-0.118-0.123) |
|  |  |  |  |  |  |  |

**Table S7.** Relative levels of metabolites in 55 faecal samples from 16 CS-delivered infants, over 30 days following birth.

Significant difference in metabolite levels (p<0.05) are reported between day 1 and 2 (^a^), 1 and 3 (^b^), 1 and 7 (^c^), 1 and 15 (^d^), 1 and 30 (^e^), 2 and 3 (^f^), 2 and 7 (^g^), 2 and 15 (^h^), 2 and 30 (^i^), 3 and 7 (^j^), 3 and 15 (^k^), 3 and 30 (^l^), 7 and 15 (^m^), 7 and 30 (^n^), 15 and 30 (^o^), as obtained by One-way ANOVA and Kruskal Wallis test.
